# Defining the landscape of circular RNAs in neuroblastoma unveils a global suppressive function of MYCN

**DOI:** 10.1038/s41467-023-38747-4

**Published:** 2023-07-04

**Authors:** Steffen Fuchs, Clara Danßmann, Filippos Klironomos, Annika Winkler, Jörg Fallmann, Louisa-Marie Kruetzfeldt, Annabell Szymansky, Julian Naderi, Stephan H. Bernhart, Laura Grunewald, Konstantin Helmsauer, Elias Rodriguez-Fos, Marieluise Kirchner, Philipp Mertins, Kathy Astrahantseff, Christin Suenkel, Joern Toedling, Fabienne Meggetto, Marc Remke, Peter F. Stadler, Patrick Hundsdoerfer, Hedwig E. Deubzer, Annette Künkele, Peter Lang, Jörg Fuchs, Anton G. Henssen, Angelika Eggert, Nikolaus Rajewsky, Falk Hertwig, Johannes H. Schulte

**Affiliations:** 1 Department of Pediatric Oncology and Hematology, Charité - Universitätsmedizin Berlin, 13353 Berlin, Germany; 2 The German Cancer Consortium (DKTK), Partner Site Berlin, 10117 Berlin, Germany; 3 The German Cancer Research Center (DKFZ), 69120 Heidelberg, Germany; 4 Berlin Institute of Health at Charité – Universitätsmedizin Berlin, 10178 Berlin, Germany; 5 CRCT, Inserm, CNRS, Université Toulouse III-Paul Sabatier, Centre de Recherches en Cancérologie de Toulouse, Université de Toulouse, 31037 Toulouse, France; 6 Laboratoire d’Excellence Toulouse Cancer-TOUCAN, 31037 Toulouse, France; 7 Bioinformatics Group, Department of Computer Science, and Interdisciplinary Center for Bioinformatics, University of Leipzig, 04107 Leipzig, Germany; 8 Department of Genome Regulation, Max Planck Institute for Molecular Genetics, 14195 Berlin, Germany; 9 Experimental and Clinical Research Center (ECRC) of the Charité and Max-Delbrück-Center for Molecular Medicine (MDC) in the Helmholtz Association, 13125 Berlin, Germany; 10 Core Unit Proteomics, Berlin Institute of Health at Charité - Universitätsmedizin Berlin and Max Delbrück Center for Molecular Medicine (MDC), 13125 Berlin, Germany; 11 Systems Biology of Gene Regulatory Elements, Berlin Institute for Medical Systems Biology, Max Delbrück Center for Molecular Medicine in the Helmholtz Association, Hannoversche Straße 28, 10115 Berlin, Germany; 12 Department of Pediatric Oncology, Hematology and Clinical Immunology, Heinrich Heine University Düsseldorf, Medical Faculty, and University Hospital Düsseldorf, 40225 Düsseldorf, Germany; 13 The German Cancer Consortium (DKTK), Partner Site Essen/Düsseldorf, 40225 Düsseldorf, Germany; 14 Institute of Neuropathology, Heinrich Heine University Düsseldorf, Medical Faculty, and University Hospital Düsseldorf, 40225 Düsseldorf, Germany; 15 Department of Pediatric Oncology, Helios Klinikum Berlin-Buch, 13125 Berlin, Germany; 16 Department I - General Pediatrics, Hematology/Oncology, University Children’s Hospital, Eberhard Karls University Tuebingen, 72076 Tuebingen, Germany; 17 Department of Pediatric Surgery and Pediatric Urology, University Children’s Hospital, Eberhard Karls University Tuebingen, 72076 Tuebingen, Germany; 18 Present Address: Lonza Drug Product Services, 4057 Basel, Switzerland

**Keywords:** Paediatric cancer, Paediatric cancer

## Abstract

Circular RNAs (circRNAs) are a regulatory RNA class. While cancer-driving functions have been identified for single circRNAs, how they modulate gene expression in cancer is not well understood. We investigate circRNA expression in the pediatric malignancy, neuroblastoma, through deep whole-transcriptome sequencing in 104 primary neuroblastomas covering all risk groups. We demonstrate that *MYCN* amplification, which defines a subset of high-risk cases, causes globally suppressed circRNA biogenesis directly dependent on the DHX9 RNA helicase. We detect similar mechanisms in shaping circRNA expression in the pediatric cancer medulloblastoma implying a general MYCN effect. Comparisons to other cancers identify 25 circRNAs that are specifically upregulated in neuroblastoma, including circARID1A. Transcribed from the *ARID1A* tumor suppressor gene, circARID1A promotes cell growth and survival, mediated by direct interaction with the KHSRP RNA-binding protein. Our study highlights the importance of MYCN regulating circRNAs in cancer and identifies molecular mechanisms, which explain their contribution to neuroblastoma pathogenesis.

## Abstract (web summary)

The role of circular RNAs (circRNAs) in modulating gene expression in cancer remains to be investigated. Here, the authors perform whole transcriptome sequencing in 104 primary neuroblastomas and reveal the involvement of MYCN in regulating circRNAs.

## Introduction

This regulatory RNA class was largely neglected in transcriptome analysis until 2012/2013, when circular RNAs (circRNAs) were discovered to be ubiquitously expressed, highly conserved and involved in gene regulation^1–3^. circRNAs are generated by non-canonical splicing, termed back-splicing. Their ring-like structure lacking 5’ and 3’ termini renders them exonuclease resistant and more stable than linear RNAs. Emerging findings from investigations into possible function of selected circRNAs implicate interaction with microRNAs^4^ and RNA-binding proteins (RBPs)^3,5,6^ or coding for protein products^7^ to regulate gene expression^8^. Thus, circRNAs could directly influence the biology of cancers, as described by several reports linking deregulated expression of selected circRNAs to different cancer hallmarks^9^. Generation of circRNAs is tissue-specific and regulated by RBPs^10^. Exosomes secreted from cancer cells and linked to metastatic niche preparation have been reported to contain circRNAs^11,12^, suggesting circRNA can influence nonmalignant cells to impact disease pathology. circRNAs are most abundant in neural tissues, with tightly regulated expression following neuronal differentiation^13^, suggesting their potential relevance in the pathogenesis of the pediatric cancer, neuroblastoma, arising from peripheral neuron precursor cells.

Neuroblastoma is an embryonal tumor with broadly molecularly heterogeneous tumor biology and clinical presentation^14^. While some neuroblastomas spontaneously regress without treatment, ~50% of patients present with high-risk disease at diagnosis, and only 30–40% survive five years^15^. *MYCN*, amplified in ~20% of primary neuroblastomas and determining high risk for clinical treatment stratification^16^, is the most important oncogenic driver of neuroblastoma identified to date. Recent molecular-based risk classification adds *ATRX* mutations and *TERT* rearrangements as further genomic aberrations defining high risk^17^. Messenger RNA expression has been extensively investigated in neuroblastoma, while investigations of noncoding RNAs have primarily been limited to miRNAs^18^ and lncRNAs^19^ (reviewed in ref. ^20^).

Here we extend the quantification of the neuroblastoma transcriptome to include circRNAs. An unbiased sequencing approach was applied to a large sample cohort of 104 primary neuroblastomas representing all clinically defined risk groups. We reveal a suppressive function of MYCN on circRNA biogenesis in high-risk neuroblastoma. This function is linked to a direct regulation of the DHX9 RNA helicase. Comparison of our neuroblastoma dataset with RNA sequencing data from other cancers and controls identified circRNAs specifically upregulated in neuroblastoma, of which circARID1A controlled neuroblastoma cell survival and proliferation. Interaction studies discovered KHSRP as an important RBP mediating circARID1A function. Our work provides evidence on the significance of circRNAs for neuroblastoma pathogenesis and extends knowledge about MYCN function, which implies broad relevance to cancers with an alteration of this oncogene.

## Results

### Quantifying circRNA expression in neuroblastoma

**Fig. 1:**
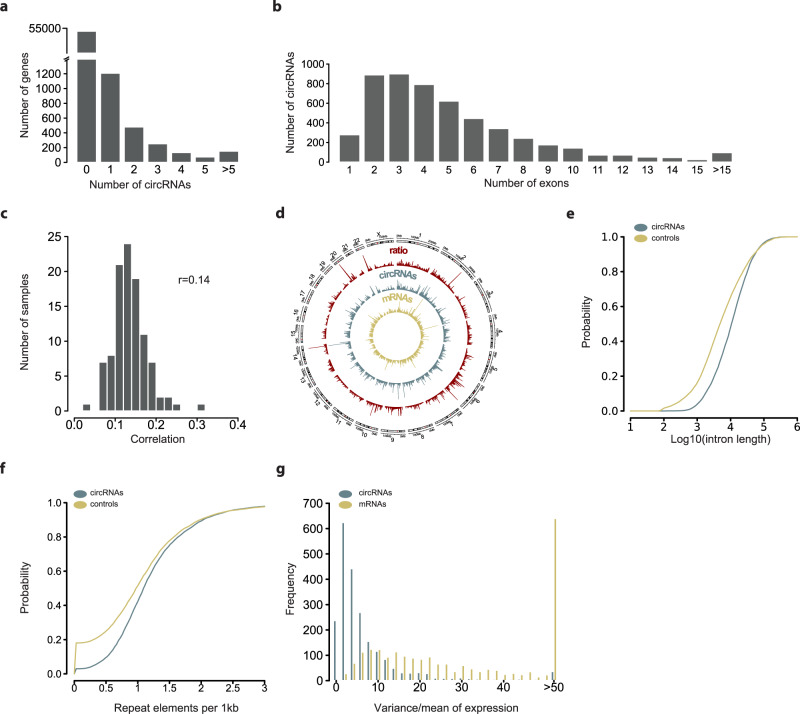
Features of detected circular RNAs in neuroblastoma. **a**–**g** Total RNA sequencing of 104 independent neuroblastoma samples was performed to detect circRNAs. **a** Number of circRNA isoforms per gene. **b** Number of exons per detected circRNA. **c** Distribution of spearman correlations of global circRNA expression with cognate mRNA expression per tumor sample. Mean correlation is shown. **d** Genome-wide map of circRNA isoform (blue) and mRNA isoform (yellow) expression per chromosome as a Circos plot. The ratio of circRNA isoforms and mRNA isoforms (red) is shown. **e** Distribution of flanking intron length of exons of genes producing circRNAs (blue) in comparison to controls (olive). **f** Distribution of repeat elements in flanking introns of exons of genes producing circRNAs (blue) in comparison to controls (olive). **g** Distribution of the ratio of variance:mean of transcript expression for circRNA (blue) and mRNA (olive) expression across the tumors. Source data are provided as a Source Data file.

Neuroblastoma samples were gathered from all risk groups to represent the entire clinical disease spectrum (Supplementary Data 1). We applied transcriptome sequencing to each of the 104 patient samples. The sequencing approach was designed to simultaneously profile circRNAs and messenger RNAs, including those transcribed from the same loci as circRNAs. Total RNA sequencing libraries generated on average 116.3 million raw reads per library and 72.7% of each sample, on average, mapped to the genome (Supplementary Fig. 1). Following standard protocols, reads were also mapped to possible back-splice junctions. This procedure identified 39,841 putative circRNAs. As expected, they were primarily generated from exons (33,459 compared to 4244 intron-derived reads and 2138 intergenic reads). To only detect robustly expressed circRNAs, we used expression filters (>20 back-splice junction-reads in >3 samples and/or expression in >25% of samples), resulting in 5203 unique putative circRNAs (Supplementary Data 2). Of the top highly expressed circRNAs, we randomly picked 10 for further validation. Of those, 7 were more resistant to exonuclease treatment than the corresponding host messenger RNA. After blocking transcription with actinomycin D, 9 of 10 were also more stable compared to host RNAs (Supplementary Fig. 1). Consistent with the literature^3^, these data suggest that the majority of our detected 5203 circRNAs are true circRNAs. Our data indicate that 2302 genes across the entire neuroblastoma transcriptome express circRNAs, of which most genes produce one predominant circRNA isoform (Fig. 1a). Most acceptor exons (93.5%) were in coding sequences of protein-coding genes, with a small minority located in 3’UTRs (1.4%) or noncoding regions (5.1%). In general, circRNAs harbored few exons, with only 2–4 exons for the majority of candidates (Fig. 1b). Abundance of a circRNA was generally lower, but only weakly correlated with mRNA expressed by its host gene in a given tumor sample (*r* = 0.14; Fig. 1c), implicating independent circRNA biogenesis.

We sought to identify genomic loci producing more circular than linear transcripts. The ratio of circRNA to mRNA isoforms was >1.2 for 45 genes (Fig. 1d, Supplementary Fig. 1, Supplementary Data 3), which included *XRN2* (ratio: 5), *GRK3* (ratio: 4) and *NBAS* (ratio: 1.55). The circRNAs expressed in each tumor sample were stratified by whether they originated from a gene with a ratio of circRNA/mRNA isoforms >1.2, which we termed *circRNA-productive genes*. Whole-genome sequencing data (available for 6*4*/104 patients, Supplementary Fig. 1) were used in integrative analyses to determine whether genomic aberrations creating copy-number changes were more common in areas of *circRNA-productive genes*. Most circRNAs were generated from copy-number neutral regions, and with no significant difference in frequencies of genes producing few or many circRNAs. Only genomic regions with a copy-number loss contained slightly fewer *circRNA-productive genes* (*p* < 0.05). Our data support that high circRNA expression from single genes does not result from underlying genomic aberrations.

The introns flanking circRNA-producing exons were longer (mean: 22,383.94 bp) than introns not involved in circRNA production (mean: 18,862.01 bp, Fig. 1e), and enriched with low-complexity regions and repetitive (e.g. Alu) elements (*p* < 1e−16; Fig. 1f) in accordance with the reported association of repetitive elements and long introns with circRNA biogenesis^2^.

The median length of all detected circRNAs predicted by short-read sequencing was 844 nt (728 nt when only considering exonic circRNAs). We aimed to evaluate this estimation by using our recently developed protocol for the Oxford Nanopore long-read sequencing platform to sequence circRNAs in full length^21^. We sequenced six different neuroblastoma cell lines and detected a shorter median length of 474 nt (Supplementary Fig. 1), which is in the range of previous studies^22,23^.

We further detected less variable expression of circRNAs compared to mRNAs across the neuroblastoma samples, indicating a more tightly regulated expression (*p* < 1e−16; Fig. 1g). Together, our results identify circRNAs as a globally and stably expressed class of transcripts transcriptionally independent of their cognate mRNAs in neuroblastoma.

### circRNA expression in neuroblastoma correlates with high clinical risk and *MYCN* amplification

**Fig. 2:**
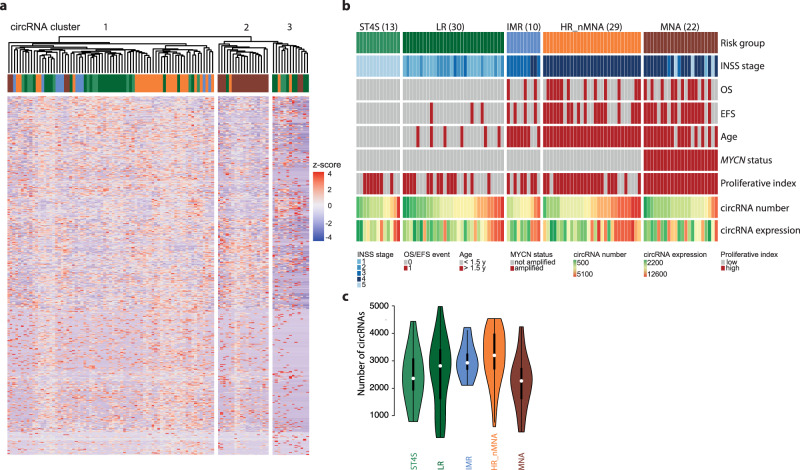
Expression of circRNAs is associated with neuroblastoma risk groups. **a** Distinct expression clusters revealed by hierarchical clustering of 104 independent neuroblastoma samples based on circRNA expression (color code of samples as in panel **b**). **b** Key clinical and biological characteristics of the analyzed 104 independent neuroblastoma patients categorized in the different risk groups shown as an Oncoplot. Number of patients in parenthesis. Proliferative index is based on a transcriptional signature. ST4S stage 4S, LR low risk, IMR intermediate risk, HR_nMNA high-risk non *MYCN*-amplified, MNA *MYCN-*amplified, INSS International Neuroblastoma Staging System, OS overall survival, EFS event-free survival. **c** Number of unique circRNA isoforms identified per risk group of the 104 independent neuroblastoma patients. Data are presented as violin plot. Violin plots use normal optimal smoothing. The median (white dot), quartiles (box), and 1.5-fold interquartile range (whiskers) are displayed. Source data are provided as a Source Data file.

We investigated whether distinct circRNA expression patterns are associated with different risk groups as defined by the International Neuroblastoma Risk Group (INRG)^24,25^. Hierarchical clustering revealed three principal clusters based on individual circRNA abundance (Fig. 2a). ‘Cluster 2’ consisted almost entirely of samples from high-risk cases with *MYCN* amplifications, supporting a distinct circRNA expression profile in this subgroup. Association of clinicopathological features, such as patient risk group with circRNA number/expression were investigated. High-risk cases lacking *MYCN* amplifications had the highest circRNA abundance and expression, whereas the lowest circRNA abundance and expression occurred in tumors harboring *MYCN* amplifications (Fig. 2b,c; Supplementary Fig. 2). Of note, *c-MYC*, a paralog of *MYCN*, was almost not expressed in our neuroblastoma cohort, independent of the risk-group (Supplementary Fig. 2). Since circRNAs were reported to be less abundant in proliferative tissues such as cancer samples^26^, we assessed for each patient sample a proliferative index using a published proliferation-associated expression signature^27^. *MYCN* amplifications slightly increased the sample proliferative index (*p* = 0.014, Fig. 2b, Supplementary Fig. 2). However, circRNA downregulation was not primarily due to proliferation in these tumors, since comparisons of only the most proliferative high-risk samples still showed that circRNA expression decreased in samples with *MYCN* amplifications (*p* < 1e−16, Supplementary Fig. 2). Our data highlight distinct circRNA expression profiles in risk groups, wherein circRNAs are least abundant in high-risk neuroblastomas harboring *MYCN* amplifications.

### MYCN globally represses circRNA expression

**Fig. 3:**
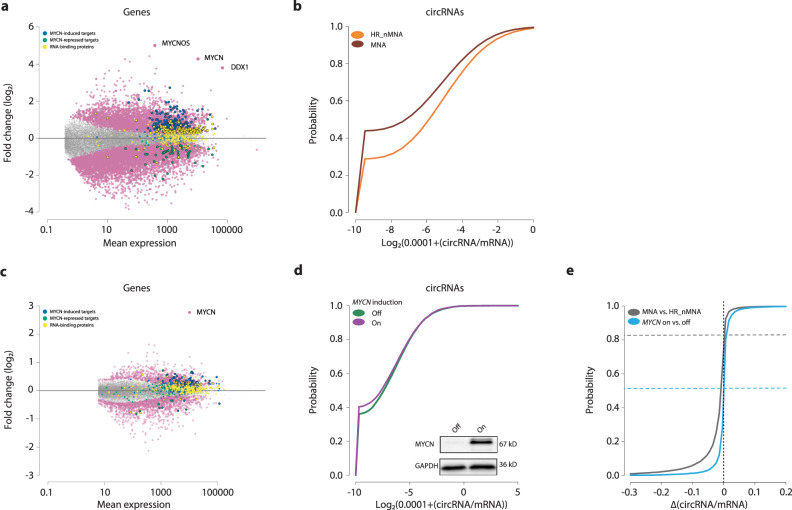
MYCN globally represses circRNA expression in neuroblastoma. **a** Differential gene expression analysis between *MYCN*-amplified (MNA, *n* = 22 biologically independent samples) and non-*MYCN* amplified (HR_nMNA, *n* = 29 biologically independent samples) high-risk neuroblastoma tumors based on the generated total RNA sequencing data. Reported are detected RNA-binding proteins (yellow), published induced (blue) and repressed (green) MYCN targets. Significant RBPs and MYCN targets are marked in large circles, non-significant ones in small circles. **b** Distribution of the circular to linear read-count ratios from **a** in the MNA tumors in comparison to HR_nMNA tumors. Mann–Whitney U test, two-sided, *p* < 1e−16. **c** SK-N-AS neuroblastoma cells harboring an inducible *MYCN*-expression system were induced for 120 h and a similar differential expression analysis was performed as in **a** in comparison to a control treatment (*n* = 3 biologically independent experiments). **d** Distribution of the circular to linear read-count ratios from **c** in the induced cells (On) in comparison to control condition (Off). MYCN induction was confirmed by western blot (*n* = 3 biologically independent experiments). Mann–Whitney U test, two-sided, *p* = 1.3e−7. **e** Distributions of the affected circRNA/mRNA ratios in MNA vs HR_nMNA tumors from **b** and SK-N-AS MYCN-induced vs. control cells from **d**. Shown is the overlap of the datasets. Source data are provided as a Source Data file.

We explored the link between *MYCN* amplification and global circRNA downregulation in more detail by comparing differentially expressed genes in high-risk neuroblastomas with or without *MYCN* amplification. This revealed 4212 upregulated and 6616 downregulated genes (Fig. 3a; Supplementary Data 4). *MYCN* and its associated neighboring genes, *MYCNOS* and *MYCNUT*, were among the most upregulated genes. Published targets activated and repressed by MYCN were significantly enriched (*p* < 1e−16) among up- and downregulated genes, respectively (Fig. 3a). A large number of RBPs, including splicing factors, were differentially expressed (82 up, 17 down; Fig. 3a). Among a global circRNA downregulation (408 circular transcripts) in *MYCN*-amplified samples, 25 circRNAs were upregulated, including CDR1-AS, the earliest studied circRNA in cancer (Fig. 3b, Supplementary Data 4). While expression of genes producing circRNAs was reduced in *MYCN*-amplified tumors (*p* < 1e−16; Supplementary Fig. 4), circRNA:mRNA ratios were also significantly lower (*p* < 1e−16), indicating that oncogenic MYCN levels had a stronger negative impact on circRNA biogenesis than general MYCN-dependent transcriptional control.

**Fig. 4:**
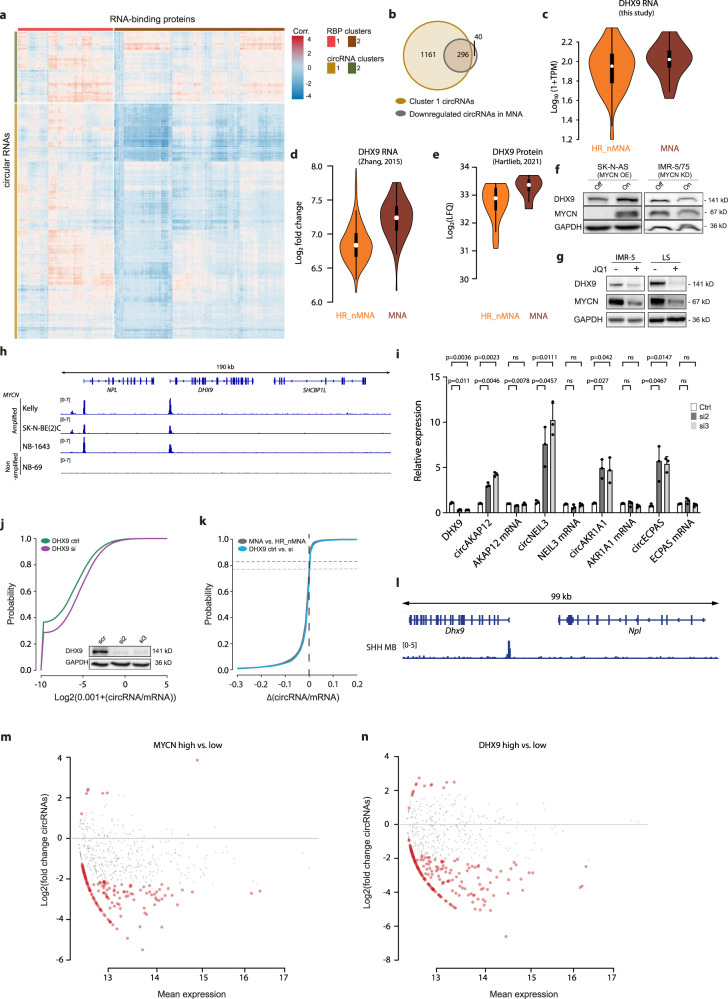
MYCN controls the RNA helicase DHX9 to suppress circRNA expression. **a** Hierarchical clustering of spearman correlations of the expression of RNA-binding proteins (RBP) and circRNAs in neuroblastoma tumor samples (*n* = 104 biologically independent samples). **b** Overlap of circRNAs downregulated in *MYCN*-amplified (MNA) tumors with the circRNAs of cluster 1. **c**–**e** Expression of DHX9 in high-risk non MYCN-amplified neuroblastoma (HR_nMNA) vs MNA of biologically independent patient cohorts. **c** Expression of DHX9 RNA in the cohort of this study (HR_nMNA *n* = 29, MNA *n* = 22); **d** Expression of DHX9 RNA in the published cohort of Zhang et al.^34^ (HR_nMNA, *n* = 401, MNA *n* = 92); **e** Abundance of DHX9 protein was determined in published mass spectrometry data of the published cohort by Hartlieb et al.^35^ (HR_nMNA *n* = 22, MNA *n* = 12). Data in **c**–**e** are presented as a violin plot. Violin plots use normal optimal smoothing. The median (white dot), quartiles (box), and 1.5-fold interquartile range (whiskers) are displayed. **f** Abundance of DHX9 was determined by western blot after induction of MYCN in SK-N-AS cells with an inducible expression system (*n* = 3 biologically independent experiments) or knockdown of MYCN in IMR-5/75 neuroblastoma cells with an inducible shRNA targeting MYCN (*n* = 3 biologically independent experiments). **g** IMR-5 and LS neuroblastoma cells were treated with the BET bromodomain inhibitor JQ1 to inhibit MYCN and protein abundance of MYCN and DHX9 was observed by western blot (*n* = 3 biologically independent experiments). **h** Analysis of published^56,82^ MYCN ChIP sequencing data from 3 different *MYCN*-amplified neuroblastoma cell lines (Kelly, SK-N-BE(2)C, NB-1643) and 1 non-amplified cell line (NB-69). **i** Expression of DHX9 together with circRNAs and cognate mRNAs determined by qRT-PCR after DHX9 knockdown with 2 different siRNAs in comparison to a scrambled control in IMR-5 cells (*n* = 3 biologically independent experiments, Data are presented as mean ± SD, Two-way ANOVA test). **j** Distribution of the circular to linear read-count ratios after DHX9 knockdown in IMR-5 cells (2 different siRNAs) in comparison to control (*n* = 3 biologically independent experiments). The knockdown was confirmed by western blot (insert). Mann–Whitney U test, two-sided, *p* < 1e−16. **k** Distributions of the affected circRNA/mRNA ratios in MNA vs HR_nMNA tumors from **3b** and DHX9 knockdown vs. control cells from **j**. Shown is the overlap of the ratios. **l** Analysis of published^38^ MYCN ChIP sequencing data from murine medulloblastoma tumorspheres (*n* = 1). Analysis of published 39 total RNA sequencing data of medulloblastomas (*n* = 39 biologically independent samples) and effect on circRNA expression, **m**, in high vs. low *MYCN*-expressing samples, **n**, in high vs. low *DHX9*-expressing samples. Source data are provided as a Source Data file.

To dissect whether circRNA downregulation was attributable to high MYCN levels, we analyzed the direct effect of MYCN induction on circRNA expression in a neuroblastoma cell model derived from the SK-N-AS cell line^28^, which lacks *MYCN* amplification. Tetracycline treatment inducing MYCN to oncogenic levels (Fig. 3d, Supplementary Fig. 3) strongly upregulated expression of *MYCN* and associated target genes (*p* = 3e−3, Fig. 3c) and 20 RBPs (among 1322 genes) while it downregulated 5 RBPs (among 1355 genes). In line with findings from patient samples, inducing MYCN significantly reduced circRNA:mRNA ratios (*p* = 1.3e−7, Fig. 3d), but did not significantly impact proliferation as described in the literature^29,30^ (assessed by proliferation index, *p* = 0.5; and measured in vitro in real-time, *p* = 0.3; Supplementary Fig. 3). As a further confirmation of the MYCN effect, we employed a cell model based on *MYCN*-amplified IMR-5/75 cells that allowed an inducible *MYCN* knockdown (Supplementary Fig. 3, Fig. 4f). Consequently, after knockdown induction, we detected a global upregulation of circRNAs (*p* < 2e−16).

Comparing data from the SK-N-AS cell model with induced *MYCN* expression and *MYCN*-amplified tumor samples detected 4253 commonly expressed circRNAs. Among the downregulated circRNAs (reduced circRNA:mRNA ratios) in the *MYCN-*amplified tumors, we found 52% of circRNAs as well to be suppressed in the cell model (Fig. 3e). Vice versa, 83% of ratio reductions in the cell model were detected in the high-risk tumors. The most downregulated circRNAs in the cell model were highly enriched among the top 500 downregulated circRNAs in *MYCN*-amplified neuroblastomas (*p* < 1e−16), thus showing in total that the MYCN-inducible cell model was able to reproduce the findings in the patient tumors.

Since oncogenic MYCN levels are known to amplify global transcription^31^, we sought to verify that the observed reduction in circRNA:mRNA ratios was not due to rising mRNA levels. Downregulation of four circRNAs selected among the most downregulated circRNAs from our RNA sequencing data was confirmed by qRT-PCR from absolutely quantified equal numbers of induced:uninduced SK-N-AS cells with expression normalized to spike-in controls (of known concentration and sequence) from the *External RNA Control Consortium* (Supplementary Fig. 4). Together our results support that MYCN globally downregulates circRNA levels in neuroblastoma.

### MYCN controls the DHX9 RNA helicase to globally suppress circRNA expression

We next aimed to decipher how MYCN exerts a global suppressive effect on circRNA abundance. RBPs are currently considered the primary factors exerting control on circRNA biogenesis^32^. We hypothesized that aberrant RBP regulation by MYCN could result in circRNA suppression. Indeed, several RBPs including splicing factors were differentially expressed with oncogenic MYCN levels in patient samples and our cell model, suggesting a MYCN-dependent effect via one or several of these factors on circRNA biogenesis. To identify RBPs potentially regulating circRNA biogenesis, we hierarchically clustered circRNA-correlated RBP expression, revealing two distinct RBP and circRNA clusters (Fig. 4a). The majority of circRNAs in the larger ‘cluster 1’ were weakly correlated or anticorrelated with ‘cluster 2’ RBP expression. The circRNA ‘cluster 1’ was also strongly enriched with circRNAs downregulated in *MYCN*-amplified tumor samples (*p* < 1e−8, Fig. 4b). DExH-box helicase 9 (*DHX9*) was among RBPs that only weakly correlated with most ‘cluster 1’ circRNAs (*r* = 0.2163). The DHX9 RNA helicase was recently demonstrated to suppress circRNA biogenesis in HEK293 cells by binding to Alu repeats^33^. Interestingly, *DHX9* was among RBPs upregulated in *MYCN*-amplified high-risk neuroblastoma samples of our cohort (*p* = 5e−3, Fig. 4c) and of an independent published cohort^34^ that we re-analyzed (92 with and 401 lacking *MYCN* amplifications, *p* < 1e−16, Fig. 4d). Upregulation of DHX9 protein in high-risk neuroblastomas contingent on *MYCN* amplification was confirmed by re-analyzing a published mass spectrometry data set^35^ from 34 (12 with and 22 lacking *MYCN* amplifications) independent neuroblastoma samples (*p* = 0.01, Fig. 4e). Accordingly, DHX9 levels were elevated in SK-N-AS cells after induction of *MYCN* expression and reduced upon *MYCN* knockdown in IMR-5/75 cells (Fig. 4f). For additional confirmation, we treated two *MYCN*-amplified neuroblastoma cell lines, IMR-5 and LS, with the BET bromodomain inhibitor JQ1 in order to inhibit MYCN (Fig. 4g). Consequently, this treatment downregulated both, MYCN and DHX9 levels, which further indicates the regulation of DHX9 by MYCN.

Since MYCN is a transcription factor, we analyzed publicly available MYCN ChIP sequencing data from three neuroblastoma cell lines harboring *MYCN* amplifications and one cell line without an amplification. The strong signal at the DHX9 promoter detected only in *MYCN*-amplified cell lines (Fig. 4h) supports direct *DHX9* regulation by MYCN. DHX9 knockdown (validated on RNA and protein levels, Fig. 4i, j) upregulated several circRNAs (qRT-PCR of selected circRNAs, Fig. 4i), while expression of the corresponding cognate mRNAs was unaffected or even reduced. Moreover, circRNAs reported as not regulated by DHX9 in HEK293 cells^33^ were unaffected by *DHX9* knockdown in IMR-5 cells (Supplementary Fig. 5). To investigate the specific binding of DHX9 to circRNAs with Alu repeat-enriched flanking introns, we performed RNA immunoprecipitation with an antibody targeting DHX9 in IMR-5 cells (Supplementary Fig. 5). qRT-PCR targeting flanking intron sequences enabled detection of a specific enrichment of the same circRNAs upregulated after *DHX9* knockdown (Supplementary Fig. 5, Fig. 4i). Further, to evaluate the effect of modified DHX9 levels on RNA circularization, we employed as a reporter assay a vector that harbors a nonfunctional split *GFP*, which is flanked by intronic sequences with complementary Alu elements^36^ (Supplementary Fig. 5). Pairing of the intronic sequences promotes back-splicing and produces a functional GFP, while binding of DHX9 to the Alu elements inhibits this process. DHX9 knockdown, (confirmed by western blot) in SH-EP cells transfected with the vector, led to an increase in GFP positive cells (Supplementary Figs. 5 and 6). MYCN inhibition with JQ1 in *MYCN*-amplified IMR-5 cells led to downregulation of MYCN and DHX9 levels, as indicated by western blot, and an increase in GFP positive cells (Supplementary Figs. 5 and 6). This implies a dose dependent effect of DHX9 on RNA circularization, which is controlled by MYCN. The generally suppressive effect of DHX9 on circRNA biogenesis implied by these results prompted us to perform RNA sequencing to analyze the global effect of DHX9 knockdown. In general, circRNA to mRNA ratios were significantly higher (*p* < 1e−16) after DHX9 knockdown, with 1047 circRNAs upregulated and only five downregulated (Fig. 4j, Supplementary Data 5). We discovered that flanking introns of the upregulated circRNAs harbored significantly more Alu repeats than those of circRNAs that were unchanged after *DHX9* knockdown (*p* = 0.002), which is in line with the current literature^33^. Approximately 78% of the globally downregulated circRNAs in *MYCN*-amplified high-risk tumor samples were upregulated by DHX9 knockdown in our cell model (Fig. 4k). Consequently, 83% of the circRNAs upregulated by DHX9 knockdown were downregulated in high-risk tumors contingent on *MYCN* amplification. The top upregulated circRNAs with the largest effect size (based on differential expression analysis after DHX9 knockdown) were strongly enriched among the top 500 downregulated circRNAs in high-risk tumor samples (*p* < 1e−16). Further, clustering our neuroblastoma patients in high and low *DHX9* expression, similarly reveals a global downregulation of circRNAs in *DHX9* high-expressing tumors (Supplementary Fig. 5). We then explored whether the MYCN-DHX9 axis could be linked to downregulation of circRNA biogenesis in another embryonal tumor, medulloblastoma, in which *MYCN* is amplified in 5–10% of cases^37^. We analyzed published MYCN ChIP sequencing data from medulloblastoma tumorspheres^38^ and detected a strong signal at the DHX9 promoter (Fig. 4l), thus indicating a direct regulation of DHX9 by MYCN also in medulloblastoma. Re-analysis of RNA sequencing data from medulloblastomas^39^ showed that circRNAs were significantly downregulated (*p* < 1e−15) in samples with high (top 20 percentile) *MYCN* expression (149 down, 7 up; *n* = 39; Fig. 4m). Classifying samples by high or low (top/lowest 20 percentile) *DHX9* expression reproduced this result (200 down, 14 up, *p* < 1e−15; Fig. 4n). Thus, oncogenic MYCN levels globally suppress circRNA expression at least in part by aberrantly upregulating the DHX9 RNA helicase, suggesting that this may be a common mechanism in MYCN-driven cancers.

### The neuroblastoma-specific circARID1A drives neuroblastoma cell proliferation and survival

**Fig. 5:**
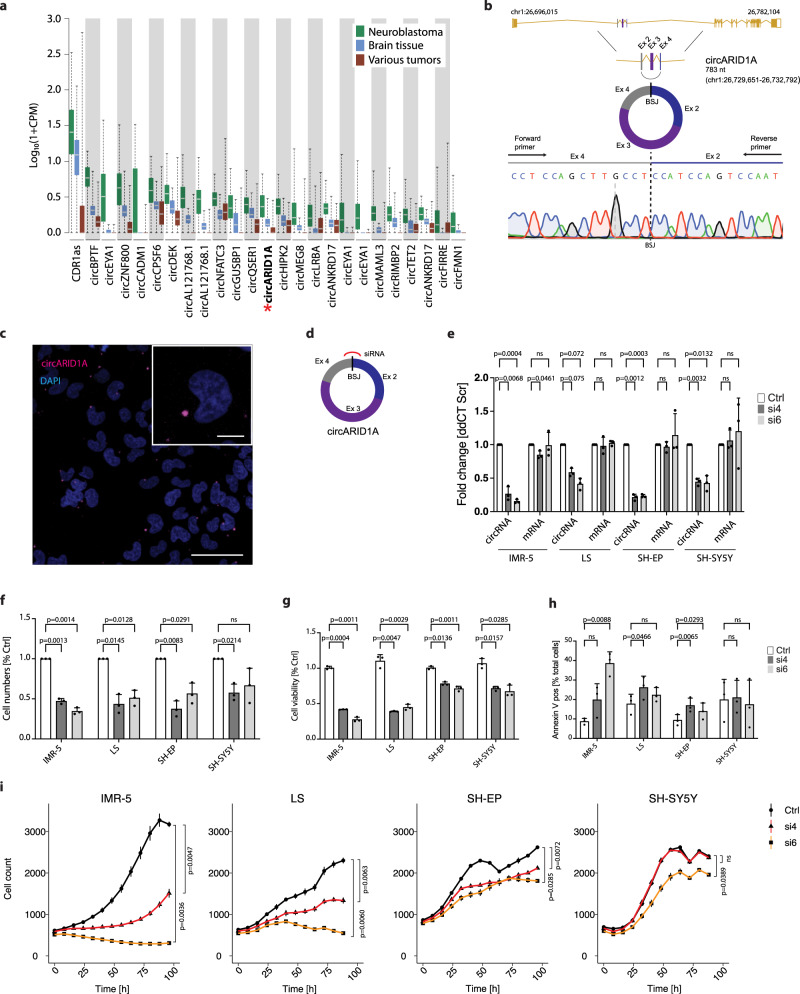
A circular RNA derived from the *ARID1A* gene drives proliferation and survival of neuroblastoma cells. **a** Comparison of circRNA expression in neuroblastoma samples (green, *n* = 104 biologically independent samples) with published normal brain tissue samples (blue, *n* = 72 biologically independent samples) and pediatric and adult cancer samples (“various tumors”, brown, *n* = 86 biologically independent samples). circARID1A is marked in bold and with an asterisk. Data are presented as a box plot. The box plot center line, violin limits and whiskers indicate the median, upper/lower quartiles and 1.5× interquartile range respectively. **b** Scheme showing the genomic representation of the *ARID1A* gene and circARID1A. The PCR detection strategy and a trace of sanger sequencing of the back-splice junction of circARID1A is shown (primers are marked by arrows). Exons (Ex) involved in circularization and the back-splice junction (BSJ) are marked. **c** Direct detection of circARID1A by RNA-FISH with a probe specific for the back-splice junction in IMR-5 cells (red color). Nuclei were stained by DAPI (blue color). Insert was digitally magnified. Scale large image 50 µm, insert 10 µm (*n* = 2 biologically independent experiments). **d** Scheme illustrating the strategy for a knockdown of circARID1A by using siRNAs targeting the BSJ. **e**–**i** Knockdown of circARID1A with 2 different siRNAs in comparison to a scrambled control in 4 different neuroblastoma cell lines (*n* = 3 biologically independent experiments, in **e**–**h** data are presented as mean ± SD, Two-way ANOVA test). **e** Expression of circARID1A and *ARID1A* mRNA as determined by qRT-PCR; **f** Total cell numbers; **g** Cell viability; **h** Annexin V positive cells as determined by flow cytometry; **i** Measurement of cell proliferation detected in real-time (Data are presented as median ± range). Source data are provided as a Source Data file.

While *MYCN* amplification is an accepted driver of adverse clinical outcome in patients with neuroblastoma, the molecular features defining high-risk neuroblastomas without *MYCN* amplification are not clearly understood. We hypothesized that circRNAs upregulated in the latter neuroblastoma subgroup may exert potential tumor-promoting functions. To test this hypothesis, we compared our total RNA sequencing data from neuroblastomas with publicly available datasets from different pediatric and adult malignancies and healthy brain tissue. This approach yielded 25 candidate circRNAs with higher expression specific to neuroblastoma, which we refer to as “neuroblastoma-specific circRNAs” (Fig. 5a, Supplementary Data 6). Among these was circARID1A, a circRNA derived from the *ARID1A* tumor suppressor gene (cirbase.org identifier^40^ hsa_circ_0008494), which was expressed more strongly in high-risk neuroblastoma samples that lacked *MYCN* amplifications (*p* < 1e−3; Supplementary Fig. 7). As *ARID1A* is recurrently mutated in neuroblastoma and associated with reduced survival^41^, we further characterized circARID1A function. A back-splice junction between *ARID1A* exon 4 and 2 (locus: chr1:26729651-26732792; Fig. 5b) generates circARID1A, with a predicted length of 783 nt. We validated the existence of a back-splice junction in circARID1A by RT-PCR and Sanger sequencing (Fig. 5b). PCR amplification of the entire RNA circle revealed that exon 3 joins exons 2 and 4 in the mature circARID1A transcript (Supplementary Fig. 7), which was also visualized by northern blotting RNA from IMR-5 and LS neuroblastoma cells and confirmed the predicted length (Supplementary Fig. 7). circARID1A proved resistant to exonuclease treatment and more stable than its cognate mRNA to degradation after actinomycin D-mediated transcription inhibition (Supplementary Figs. 1 and 7). RNA-FISH localized circARID1A (1–3 signals per cell) to the cytoplasm, which was supported by qRT-PCR from cytoplasmic cell fractions (Fig. 5c, Supplementary Fig. 7). Notably, RNA FISH of *ARID1A* mRNA showed 2- to 3-fold more signals per cell compared to circARID1A (Supplementary Fig. 7). Profiling in a panel of 11 neuroblastoma cell lines demonstrated circARID1A to be expressed throughout, but generally at lower levels than *ARID1A* mRNA (Supplementary Fig. 7). Our findings confirm the circular character of circARID1A and its cytoplasmic localization in neuroblastoma cells.

Knockdown of circARID1A in four different neuroblastoma cell lines, two *MYCN*-amplified (IMR-5, LS) and two non *MYCN*-amplified (SH-EP, SH-SY5Y) by two independent siRNAs directed against the back-splice junction strongly reduced circARID1A levels in comparison to a scrambled control siRNA with no significant change in *ARID1A* mRNA levels (Fig. 5d, e). Knockdown of circARID1A reduced cell numbers (Fig. 5f), viability (Fig. 5g), induced apoptosis (Fig. 5h, Supplementary Fig. 6), and reduced proliferation (Fig. 5i) in all cell lines, but did not induce apoptosis in SH-SY5Y cells. An independent approach using antisense oligonucleotides (ASO) to target circARID1A also downregulated circARID1A, while not affecting *ARID1A* mRNA, and resulted in a similarly reduced cell viability of IMR-5 cells (Supplementary Fig. 8). Specificity of siRNA-based knockdown was further validated by ectopically overexpressing circARID1A with a mutated back-splice junction, which impaired siRNA binding. Specific circARID1A overexpression was validated by qRT-PCR and northern blotting (Supplementary Figs. 7 and 8). Overexpression followed by siRNA-directed knockdown caused a smaller reduction in cell viability than in uninduced controls (Supplementary Fig. 8), partially rescuing the effect. Our data validate circARID1A as a factor maintaining actively proliferating neuroblastoma cells, strongly expressed in high-risk neuroblastomas.

### circARID1A uses the KHSRP RNA-binding protein for its function

**Fig. 6:**
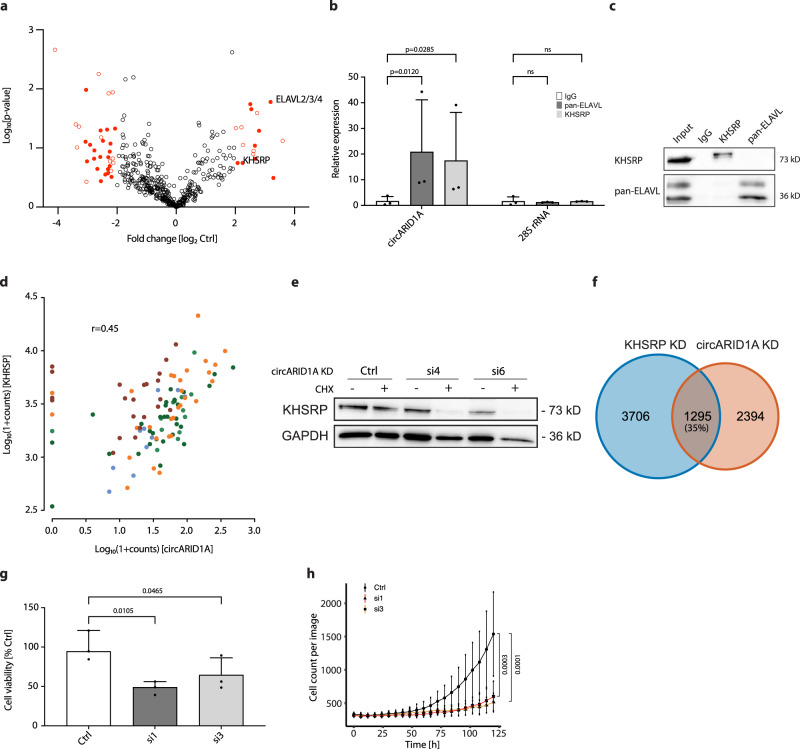
CircARID1A function is mediated by KHSRP in neuroblastoma cells. **a** Mass spectrometry of enriched proteins was performed after circARID1A-specific vs. control pulldown in IMR-5 cells (*n* = 3 biologically independent experiments). Red circles represent more than 4-fold enriched proteins. Full circles represent proteins of high confidence of which at least 3 independent peptides were detected. KHSRP and ELAVL2-4 proteins are marked. Student’s *t* test, two-sided, non-paired. **b** RNA-immunoprecipitation (RIP) specific for KHSRP and ELAVL (pan-ELAVL antibody detects all 4 ELAVL family proteins) was performed in IMR-5 cells (*n* = 3 biologically independent experiments, Data are presented as mean ± SD, Two-way ANOVA test). Anti-IgG was used as control antibody. Enrichment of circARID1A in comparison to 28S rRNA was detected by qRT-PCR. **c** Specificity of RIP in b was validated by western blot. Anti-IgG antibody served as control (*n* = 3 biologically independent experiments). **d** Spearman correlation of KHSRP and circARID1A expression in our neuroblastoma tumor cohort (*n* = 104 biologically independent samples). Color coding of risk groups as in Fig. 2b, c. **e** CircARID1A knockdown was performed with 2 different siRNAs in IMR-5 cells with or without cycloheximide (CHX) treatment (*n* = 3 biologically independent experiments). KHSRP protein levels were depicted by western blot. **f** KHSRP knockdown was performed in IMR-5 cells (2 different siRNAs vs. control, *n* = 3 biologically independent experiments; blue, left panel) and in a separate experiment a circARID1A knockdown (1 siRNA vs. control, *n* = 3 biologically independent experiments; orange, right panel) and analyzed by RNA sequencing. The number of genes detected differentially expressed in the respective dataset is reported. The two datasets were integrated and the overlap is shown in a Venn diagram. **g**–**h** KHSRP knockdown was performed in IMR-5 cells (*n* = 3 biologically independent experiments). **g** Cell viability was measured (Data are presented as mean ± SD, One-way ANOVA test); **h** Proliferation was analyzed in real time (One-way ANOVA test, data are presented as median ± range). Source data are provided as a Source Data file.

Our data suggest that neuroblastoma cells may depend on circARID1A, so we next aimed to dissect its mechanism of action. An established circRNA mode of action is via RBP interactions^8^. Mass spectrometric analysis of proteins bound to circARID1A identified 16 enriched proteins, including the RBPs, KHSRP and ELAVL2-4, in a pulldown from IMR-5 cells using a circARID1A-specific probe (vs. a scrambled probe control, Fig. 6a). Pulldown specificity was validated by qRT-PCR, which detected significant circARID1A enrichment but no enrichment of *ARID1A* mRNA or control transcripts compared to the scrambled probe (Supplementary Fig. 9). The interaction was confirmed by an independent approach co-immunoprecipitating RNA with antibodies for KHSRP or ELAVL2-4, which specifically enriched circARID1A (Fig. 6b, c). Probing circARID1A in silico for enriched RBP motifs in comparison to shuffled control sequences of the same length detected sites for 26 different RBPs, including three canonical binding sites for KHSRP but none for ELAVL or the other mass spectrometrically identified proteins (Supplementary Fig. 9), indicating direct binding only for KHSRP. Interestingly, an additional non-canonical KHSRP site (90% similarity to canonical motif) was present at the circARID1A back-splice junction, suggesting specific binding to the circRNA via this site. KHSRP expression was higher in RNA sequencing data from our neuroblastoma cohort, compared with publicly available RNA sequencing datasets from other cancers or normal brain tissue (Supplementary Fig. 9). *KHSRP* and circARID1A expression were positively correlated in neuroblastoma samples from all risk groups in our cohort (*r* = 0.45; Fig. 6d). circARID1A knockdown in IMR-5 cells reduced the KHSRP protein level without altering *KHSRP* mRNA expression (Fig. 6e, Supplementary Fig. 9). This was more prominent after blocking protein synthesis by treating cells with cycloheximide (Fig. 6e), suggesting a stabilizing function of circARID1A on KHSRP protein. We also compared differentially expressed genes in RNA sequencing data after independent knockdown of either KHSRP or circARID1A in IMR-5 cells. Genes affected by *KHSRP* knockdown made up approximately 35% of differentially expressed genes affected by circARID1A knockdown (*p* < 1e−16; Fig. 6f, Supplementary Data 7 and 8). RNAs harboring KHSRP binding motifs were significantly enriched in the up- and downregulated genes after circARID1A knockdown (*p* < 1e−16). Gene ontology terms for apoptosis induction were enriched in genes upregulated after KHSRP knockdown, while terms related to various cell cycle processes were diminished (Supplementary Fig. 9). Prompted by this result, we investigated enrichment of functionally annotated gene signatures (from the Molecular signatures database MSigDB^42^, using C2: curated gene sets) among genes upregulated upon KHSRP knockdown and identified an overrepresentation of various gene sets related to TP53 signaling (six different TP53 related gene sets among top 20). Similar to circARID1A knockdown (Fig. 5e, g, i), KHSRP knockdown in IMR-5 cells (validated on RNA and protein levels, Supplementary Fig. 9) reduced cell viability (Fig. 6g) and proliferation (Fig. 6h), substantiating the proposed functions for KHSRP in neuroblastoma cells. We present evidence for an essential interaction between circARID1A and KHSRP in neuroblastoma cells that impacts cell growth and survival.

## Discussion

Our work provides evidence for the importance of circRNAs in neuroblastoma pathology through a comprehensive analysis of the complex expression of circRNAs in neuroblastoma. We demonstrate a global suppressive effect of oncogenic MYCN levels mediated by the DHX9 RNA helicase on circRNA expression in neuroblastoma and a second childhood embryonal tumor, medulloblastoma. An essential circRNA derived from the *ARID1A* tumor suppressor gene was identified that uses the KHSRP RBP to promote neuroblastoma cell growth.

Gene mutations are intensively investigated in cancer biology, however, next-generation sequencing is most often applied to poly-A RNA, missing important information about regulatory RNAs. Our unbiased sequencing approach identified 5,203 circRNAs in samples from primary neuroblastomas and describes expression patterns across all risk groups. To date, global circRNA expression landscapes have been described for T-ALL^43^, AML^44^, mantle cell lymphoma^45^, prostate cancer^46^, cholangiocarcinoma^47^, hepatocellular carcinoma^48^, colorectal cancer^49^ and lung adenocarcinoma^50^. Vo et al. conducted a thorough study by applying unbiased, targeted exome capture sequencing to samples from 19 cancer entities^51^, reporting the tissue-specific expression of several circRNAs and the discovery of read-through circRNAs arising by circularization of read-through transcripts. This added to the understanding of global circRNA expression especially in prostate adenocarcinoma, from which the majority of surveyed samples came. Neuroblastoma samples were also analyzed, but were too few to draw global conclusions. Single circRNAs have been identified in neuroblastoma cell lines and are associated with differentiation^52^, anaerobic glycolysis^53^ or fatty acid metabolism^54^. Here we add the global circRNA expression patterns in neuroblastoma to this emerging field in cancer research.

Our analysis showed that circRNA expression was not well-correlated with mRNA expression in neuroblastoma. CircRNA biogenesis has previously been reported to be cell-type specific and independent from cognate mRNA expression^55^, occurring by an RBP-regulated switch from canonical linear splicing to alternative back-splicing^5^. We also show that circRNA expression was globally repressed in samples from neuroblastomas harboring *MYCN* amplifications, the major oncogenic driver in neuroblastoma^16^. The paralog of *MYCN*, *c-MYC*, was almost not expressed in our cohort of neuroblastoma patients and thus seemed not to significantly influence circRNA expression. Oncogenic MYCN levels are known to drive transcriptional amplification, a process by which the majority of transcriptionally active genes are further upregulated^56^. This effect poses a challenge to conventional gene expression analysis, as it renders unchanged absolute transcript levels from genes not regulated by MYCN as downregulated relative to median expression^31^. Correcting for this effect, we confirmed that oncogenic MYCN levels reduce circRNA biogenesis by normalizing transcript measurement to cell count in an inducible neuroblastoma cell model. MYCN was previously implied to regulate alternative splicing in neuroblastoma by directly controlling several splicing factors^57^. In line with this, we identified several differentially expressed RBPs in the MYCN-inducible cell model and neuroblastomas harboring *MYCN* amplifications. Hierarchical clustering of circRNA-correlated RBP expression identified the DHX9 RNA helicase as a negative regulator of circRNA expression. DHX9 was recently reported to negatively regulate circRNA biogenesis in HEK293 cells by resolving double-stranded RNA structures induced by inverted Alu repeat complementarities^33^. Introns that flank exons expressed as circRNAs have been reported to be longer than other introns, and enriched in Alu repeats^2^, also confirmed by our data. Ottesen et al. demonstrated that DHX9 knockdown in HEK293 and HeLa cells upregulated several circRNAs produced from the Alu repeat-rich *SMN* (Survival Motor Neuron) genes^58^, thus, highlighting the importance of DHX9 on circRNA biogenesis. We extend the regulatory role of DHX9 on circRNA biogenesis to a mechanism utilized by MYCN, at the very high levels reached during oncogenic activity. However, we are aware that DHX9 might not be the only RBP acting on circRNA expression since our hierarchical clustering analysis identified other candidates, too. Yet, we show that oncogenic MYCN levels in neuroblastoma act through DHX9 to globally suppress circRNA expression, and present evidence for the same activity in the related embryonal tumor, medulloblastoma, demonstrating the importance of this factor. This finding adds another regulatory layer through which oncogenic MYCN can act, which may represent a general mechanism in cancers expressing oncogenic MYCN levels.

We identified 25 circRNAs that we propose as neuroblastoma-specific, since their expression is higher in neuroblastomas than other cancers or even healthy brain tissue, which is reportedly the tissue in which circRNAs are most abundant^13^. We demonstrate that circARID1A, one of the neuroblastoma-specific circRNAs, has a cancer cell-promoting function. The host gene from which it is derived, *ARID1A*, contributes a subunit of the SWI/SNF complex, and is mutated in ~20% of human cancers^59^. *ARID1A* mutations in neuroblastoma are associated with a strongly reduced overall survival^41^. However, no *ARID1A* mutations were detected in our cohort that affected the circARID1A sequence. Interestingly, circARID1A expression was higher in high-risk neuroblastomas without *MYCN* amplifications, a subgroup in which molecular pathogenesis is less well understood. Recent reports demonstrate circARID1A expression is relevant for autism spectrum disorder and skeletal muscle cell differentiation, by interacting with microRNAs^60,61^. We demonstrate that circARID1A binds to the KHSRP RBP in neuroblastoma cells. KHSRP plays roles in pre-mRNA splicing, RNA shuttling and regulating RNA stability, particularly by binding to AU-rich elements, motifs that determine RNA stability^62^, in RNA 3’UTRs. KHSRP was recently shown to be important for circRNA biogenesis by binding to flanking introns^63^, but no direct interaction with circRNAs has been described so far. Interestingly, circARID1A knockdown destabilized KHSRP, while not affecting *KHSRP* mRNA. Consequently, a KHSRP knockdown markedly inhibited cell viability and proliferation, thus, mimicking the phenotypic changes after circARID1A knockdown. These findings support that circARID1A is important for neuroblastoma cells and exerts its function, at least in part, by interacting with KHSRP.

Here we present the global circRNA landscape in neuroblastoma, which is shaped by oncogenic MYCN mediated by the DHX9 RNA helicase. This research extends our knowledge about MYCN functionality and is likely to be applicable to other cancers with dysregulated MYCN, as exemplified for medulloblastoma. We also identify circARID1A as a neuroblastoma-associated circRNA maintaining neuroblastoma cell viability and growth at least partially through its interaction with KHSRP. Our study highlights the importance of circRNAs for neuroblastoma biology, presenting new angles for therapy design to fight high-risk disease.

## Methods

### Patients and biomaterial samples

This study was conducted in accordance with the Declaration of Helsinki and Good Clinical Practice, and informed consent was obtained from all patients or their guardians. Collection and use of patient samples was approved by the institutional review boards of *Charité - Universitätsmedizin Berlin* and the University of Cologne Medical Faculty within the trial and registry, respectively. Patients were registered and treated according to trial protocols from the German Society of Pediatric Oncology and Hematology (GPOH). Patients were enrolled in the German neuroblastoma clinical trials (NB90, NB97, NB2004) or the German Neuroblastoma Registry (NB Registry 2016)^64^. Primary tumor samples from initial biopsies were collected by the German Neuroblastoma Biobank as previously described^65^ (University of Cologne Medical Faculty) from clinical trial sites. The German Neuroblastoma Biobank provided total RNA isolated from primary tumor samples collected from 104 patients diagnosed with neuroblastoma, of which 36 were female, 67 male and 1 patient for that sex information was not available. Gender information were not available for the patients. The average age of patients was 2.4 years. Patient characteristics are reported in Supplementary Data 1. Tumor samples were staged according to the International Neuroblastoma Staging System (INSS)^66^, and patient risk was defined in accordance with the International Neuroblastoma Risk Group (INRG)^24,25^. *MYCN* copy number was determined by FISH in routine diagnostics conducted by the German Neuroblastoma Biobank. Total RNA from snap-frozen biopsied tissue with at least 60% tumor cell content, as evaluated by the trial’s reference pathologist, was isolated by the German Neuroblastoma Biobank using Trizol™ (Thermo Fisher Scientific Inc., Waltham, MA, USA) according to the manufacturer’s protocol before quantifying RNA by Qubit™ RNA Broad-Range Assay (Thermo Fisher Scientific) and determining RNA integrity using the Bioanalyzer RNA 6000 Nano instrument (Agilent Technologies, Inc., Santa Clara, CA, USA).

### Cell lines, culture and functional in vitro assays

The human neuroblastoma cell lines SK-N-BE(2)C (CRL-2268) and SK-N-FI (CRL-2142) were obtained from the American Type Culture Collection (ATCC, Manassas, VA, USA) and the cell lines Kelly (ACC-355), SH-SY5Y (ACC-209), LAN-5 (ACC-673) were obtained from the German Collection of Microorganisms and Cell Cultures GmbH (DSMZ, Braunschweig, Germany). IMR5/75 was kindly provided by F. Westermann (German Cancer Research Center, Heidelberg, Germany). IMR-5, SH-EP, SK-N-AS, SK-N-SH and GI-ME-N were kindly provided by A. Schramm (*Medizinische Fakultät, Universitätsklinikum Essen*, Essen, Germany). LS cells were kindly provided by M. Fischer (University of Cologne Medical Faculty). The MYCN-inducible cell model was previously generated^28^ from the SK-N-AS neuroblastoma cell line. Single clones were maintained in selection medium supplemented with 500 µg/ml G418-BC (Merck, Burlington, MA, USA) and 5 µg/ml blasticidin (Thermo Fisher). *MYCN* expression was induced by adding 2 µg/ml tetracycline to the medium. Induction was confirmed by qRT-PCR (*MYCN* and MYCN targets^67^, *MTHFD2* and *TERT*) and western blotting (MYCN). IMR-5/75 cells harboring an inducible expression system for an shRNA targeting *MYCN* were previously generated^68^ (kindly provided by F. Westermann, DKFZ, Heidelberg, Germany) and were cultured with 50 µg/ml zeocin (ThermoFisher Scientific) and 5 µg/ml blasticidin (ThermoFisher Scientific) for selection. Knockdown was induced by adding 2 µg/ml tetracycline to the medium and confirmed by western blotting (MYCN). Five of the used cell lines were derived from a male, and 7 from a female subject. Cell lines were authenticated by short tandem repeat DNA typing (Idexx Bioresearch, or Multiplexion), cultured for no more than 15 passages and routinely tested negative for mycoplasma contamination using the MycoAlert Mycoplasma Detection Kit (Lonza, Basel Switzerland). Cells were cultured as previously described^69^ in RPMI medium (Gibco, Thermo Fisher Scientific) supplemented with 10% fetal calf serum (FCS) and 1% penicillin/streptomycin, and incubated at 37 °C, 100% humidity and 5% CO_2_.

After harvesting with trypsin, cells were pelleted by centrifugation (500 × *g*, 5 min., 4 °C), mixed 1:1 with 0.02% Trypan blue solution (Thermo Fisher Scientific) and counted using a TC20 Automated Cell Counter (Bio-Rad Laboratories, Hercules, CA, USA). For experiments assessing transcript stability, 200,000 IMR-5/75 cells/well were seeded into 6-well plates, and treated 24 h with 4 µg/ml actinomycin D (Sigma-Aldrich, St. Louis, MA, USA) to block transcription. The non-treated control condition included DMSO at the same volume as the used drug to control from the solvent. Similarly, to assess the effects of MYCN inhibition, IMR-5 and LS cells were treated with the BET bromodomain inhibitor JQ1 (Selleckchem, Houston, TX, USA) for 2 days (IMR-5: 2 µg/ml, LS: 7.5 µg/ml). MYCN and DHX9 protein abundance were assessed afterwards by western blot. DMSO served as control treatment. To assess cell viability or proliferation, 6,000 IMR-5 cells or 4,500 SH-SY5Y cells were seeded into wells of 96-well plates and incubated under standard culture conditions. Viability was assessed using the CellTiter-Glo Luminescent Cell Viability Assay (Promega, Madison, WI, USA) according to the manufacturer’s protocol and a Glomax Multi+ Detection System luminometer (Promega, software v3.1.3). Cell proliferation was measured in real time using the IncuCyte® S3 Live Cell System (Essen Bioscience, Ann-Arbor, MI, USA). Per well, 4 photos were taken in intervals of 3–6 h through the 10x objective. Raw photos were analyzed by IncuCyte® S3 Software v2019b Rev2 (Essen Bioscience) following a cell-count approach.

### Flow cytometry to detect apoptosis

To study apoptosis induction by cARID1A knockdown in different neuroblastoma cell lines, APC-AnnexinV (Biolegend, San Diego, CA, USA) and 7-AAD viability solution (Thermo Fisher) were used according to the manufacturer. Cells were harvested using Accutase (Sigma-Aldrich) and counted to determine the cell number and viability. Stained cells were measured on a BD LSR Fortessa X-20 flow cytometer (BD Biosciences, Franklin Lakes, NJ, USA) with the BD FACS Diva software (v8.0.1) and analyzed with FlowJo software (v10.*8*). The gating strategy is shown in Supplementary Fig. 6.

### Gene/circRNA knockdown by siRNA or antisense oligonucleotides

Knockdown of circARID1A and mRNAs (ARID1A, KHSRP, DHX9) was performed using custom siRNAs (sequences listed in Supplementary Data 9) targeting the specific flanking back-splice junction or non-circularized exons, respectively. Random scrambled (or specific scrambled^70^ for circARID1A control) siRNAs were used as controls. Transfections with siRNAs targeting circARID1A were conducted in 6-well plates (assessment of expression and cell count), to which 200,000 IMR-5 cells or 150,000 SH-SY5Y cells in 1.6 ml media were pre-mixed (15 min at room temperature) with 20 µM siRNA (in 4 µl) and 4 µl Lipofectamine3000 (Thermo Fisher) in 400 µl Opti-MEM reduced-serum medium (Thermo Fisher), before incubating under culture conditions for 96 h. Transfections of siRNAs targeting circARID1A were conducted in the same way in 96-well plates (assessment of cell viability or proliferation) using 6000 IMR-5 cells or 4500 SH-SY5Y cells (in 80 µl medium per well) and 20 µM siRNA (in 0.2 µl), 0.2 µl Lipofectamine3000 and 20 µl Opti-MEM. Transfections with antisense DNA oligonucleotides targeting circARID1A were performed following the same protocol in 6-well plates (assessment of expression) or 96-well plates (assessment of cell viability or proliferation) for 3 days. Transfections of siRNAs targeting mRNAs were performed following the same protocol, but for 3 days.

### circARID1A overexpression neuroblastoma cell models

To create the constitutive circARID1A expression plasmid in the pcDNA3.1(+)Laccase2 MCS Exon Vector obtained from Addgene (a gift from Jeremy Wilusz, Addgene plasmid #69893; http://n2t.net/addgene:69893; RRID: Addgene_69893)^36^, circARID1A was amplified using the Q5 polymerase (New England Biolabs, Ipswich, MA, USA) and the circARID1A_w primer pair (sequence in Supplementary Data 9) from cDNA reverse-transcribed from total RNA isolated from IMR-5 cells. Primers were designed to add sites for the PacI and AgeI restriction enzymes 5’ and 3’ to the circARID1A sequence, respectively. The PacI/AgeI-digested fragment was purified from the agarose gel band using the QIAquick gel extraction kit, (Qiagen, Hilden, Germany) and ligated (T4 DNA ligase, New England Biolabs) to generate the pcDNA3.1_Lacc_cARID1A plasmid, which was propagated in *E. coli*.

To create a conditional circARID1A expression plasmid, the Laccase-circARID1A sequence was amplified from pcDNA3.1_Lacc_cARID1A using the Lacc_circARID1A_w primer pair (sequence in Supplementary Data 9) that respectively added XhoI and NheI restriction sites 5’ and 3’ to the Laccase-circARID1A sequence for its ligation into the Gateway™ vector, pT-REx-DEST30 (Gateway™ pT-Rex™-DEST30 Vector, Thermo Fisher). The tet-operator sequence upstream of Laccase-circARID1A allows tet-repressor binding to block transcription in the absence of tetracycline. Upon addition of tetracycline, the repressor is released and expression can occur. The pcDNA™6/TR mammalian expression vector (Thermo Fisher) was co-transfected as described previously^71^ to express the tet-repressor in the cell model.

To mutate siRNA binding sites in the pDEST_Lacc_cARID1A back-splice junction, specific primers (incorporating the desired mutations) were designed for overlap-extension PCR. Briefly, two PCR products sharing the mutant sequence were generated (primer pairs: cARID1A_w_f_PacI + cARID1A_oe_simut_r1 and cARID1A_oe_simut_f2 + cARID1A_r3_SbfI, Supplementary Data 9) that were combined in the overlap annealing reaction, to create the full-length circARID1A fragment containing PacI and SbfI restriction sites for cloning into pT-REx-DEST30. For the rescue experiment, overexpression was induced by 72 h of tetracycline treatment before performing circARID1A knockdown as described above.

### DHX9 reporter assay

To assess the effect of modified DHX9 levels on RNA circularization, SH-EP and IMR-5 cells were transfected with a plasmid harboring a nonfunctional split *GFP* (pcDNA3.1(+) ZKSCAN1 MCS-WT Split GFP+Sense IRES was a gift from Jeremy Wilusz, Addgene plasmid # 69909; http://n2t.net/addgene:69909; RRID:Addgene_69909)^36^ flanked by introns from the *ZKSCAN1* gene harboring several Alu elements, which foster circularization and render the GFP functional. Cells harboring the plasmid were selected for 48 h with 500 µg/ml G418-BC (Merck). To modify DHX9 levels, a knockdown was performed for 3 days in SH-EP cells, or MYCN was inhibited with the BET bromodomain inhibitor JQ1 in IMR-5 cells as described above. GFP positive cells were determined by flow cytometry as described above.

### circRNA validation by PCR and Sanger sequencing

RNA was isolated with Trizol™ (Thermo Scientific Fisher), reverse transcribed using the Maxima H Minus First Strand cDNA Synthesis Kit with dsDNase (Thermo Scientific Fisher) then amplified in a standard 35 cycle RT-PCR reaction performed with the Kapa Taq DNA Polymerase (Roche, Basel, Switzerland) according to the manufacturers’ instructions. Quantitative RT-PCR (qRT-PCR) utilized SYBR Green Essential Master Mix (Roche) in the StepOnePlus *real-time* PCR System (Thermo Fischer Scientific) according to manufacturer’s instructions. Data were analyzed using the StepOnePlus Software Version 2.3 (Applied Biosystems, Waltham, MA, USA). Divergent primers specific for the back-splice junction were designed for PCR assays amplifying circRNAs. Exon-spanning primers involving only exons that are not part of the circRNA were designed to amplify the cognate mRNAs. *SDHA* served as reference gene. All primer pair sequences are listed in Supplementary Data 9. To validate the back-splice junction of a circRNA, PCR products were generated using the Q5 high-fidelity polymerase (New England Biolabs) for Sanger Sequencing by Eurofins Genomics GmbH (Ebersberg, Germany) or LGC Genomics GmbH (Berlin, Germany). Circularity and resistance to exonuclease treatment by RNaseR (Lucigen, Madison, WI, USA) was validated as previously described^3^ followed by qRT-PCR to assess circRNA and mRNA levels from a host gene.

### RNA immunoprecipitation

RNA immunoprecipitation (RIP) was performed with the MAGNA RIP RNA-binding protein immunoprecipitation kit (Merck), according to manufacturer’s instructions, on pairs of 70% confluent IMR-5 cultures in 15 cm dishes per condition. Briefly, cells were UV crosslinked (254 nm and 150 mJ/cm^2^) before lysis in 100 µl complete lysis buffer/dish. A 10% input sample for western blotting (10 µl input) and RNA isolation (10 µl input + 500 µl Trizol™, Thermo Fisher Scientific) was reserved. Magnetic beads were washed before adding 5 µg of antibody. An unspecific IgG isotype antibody served as the control. Immunoprecipitation of the remaining 80 µl cell lysate was performed overnight at 4 °C on a rotating platform. Beads were washed before eluting in 15 µl loading buffer for western blotting or in 500 µl Trizol™ for RNA isolation. Western blotting was performed to test the efficiency of the immunoprecipitation. Veriblot for IP Detection Reagent (Abcam, Cambridge, UK) was used as secondary antibody to avoid interference with antibodies used for the RIP. Target enrichment relative to the control condition was quantified using qRT-PCR. To detect specific binding of DHX9 to circRNAs enriched with Alu repeats in their flanking introns, primer pairs targeting the flanking introns were designed. All antibodies and primers are listed in Supplementary Data 9.

### CircRNA pulldown assay

A circARID1A-specific pulldown was performed using 4 15 cm dishes of 70% confluent IMR-5 cultures per condition based on the method published by Theil et al.^72^ with modifications. Probes specific for the circARID1A back-splice junction (based on the siRNA-4 sequence) were designed containing a 3’-biotin-TEG. A scrambled probe served as control. Cells were UV crosslinked (254 nm and 150 mJ/cm^2^) before scraping them off in 1 ml PBS, washing and collection of cell pellets that were lysed in 1000 µl cold lysis buffer (50 mM Tris-HCl pH 7.0, 10 mM EDTA, 1% SDS) freshly supplemented with 1 mM DTT, murine RNase-inhibitor (New England Biolabs) and Complete EDTA-free Protease Inhibitor Cocktail (Roche). Lysed cells were left 30 min on ice while they were passed 5 times through a 20 G then 5 times through a 26 G needle before pelleting cellular debris (30 min centrifugation, 4 °C, maximum speed). Supernatant was mixed with 2 volumes of hybridization buffer (750 mM NaCl, 50 mM Tris-HCl pH 7.0, 10 mM EDTA, 1% SDS, 15% formamide) and split equally for RNA analysis and protein analysis (reserving 50 µl of input sample from both RNA and protein samples). Lysate was added to 50 µl of MyOne Streptavidin C1 magnetic beads (Thermo Fisher Scientific) pre-washed in lysis buffer, and incubated at 37 °C with constant rotation to preclear lysate for 1-2 h. After magnetic separation, 500 pmol of probes were added to the lysate and incubated for 2 h at 37 °C before adding to 50 µl of pre-washed magnetic beads and incubation for 1 h at 37 °C. The beads were washed 5x in wash buffer (2x SSC buffer, 0.5% SDS) and further processed for LC-MS analyses (see below). For RNA analysis 100 µl of proteinase K buffer (100 mM NaCl, 10 mM Tris-HCl pH 7.0, 1 mM EDTA, 0.5% SDS, 1 mg/ml proteinase K freshly added) was added to the beads and in parallel to the input sample and incubated at 50 °C for 45 min, and were shaken at 1300 rpm to digest proteins. Proteinase K was inactivated by incubating for 10 min at 95 °C. Then 500 µl of Trizol™ (Thermo Fisher Scientific) was added to the samples and magnetically separated from the beads following RNA isolation as described above. The enrichment of circARID1A in comparison to other transcripts was determined by qRT-PCR in comparison to the input sample. Sequences of probes are mentioned in Supplementary Data 9.

### LC-MS analysis

Denaturation buffer (6 M urea, 2 M thiourea in 50 mM HEPES, pH 8) was added to the washed beads from circRNA pulldown experiments and samples were processed and analyzed as described^73^. Briefly, proteins were reduced with 10 mM DTT and alkylated with 55 mM chloroacetamide (Sigma), digested with Endopeptidase LysC and sequence grade trypsin. Acidified peptides were cleaned-up and analyzed by reversed phase chromatography (98 min gradient of 2–55% acetonitrile) on a High-Performance Liquid Chromatography (HPLC) system (Thermo Fischer Scientific) coupled to an Q Exactive Plus mass spectrometer (Thermo Fischer Scientific). The instrument was operated in the data-dependent mode with performing full scans (70 K resolution; 3 ×10^6^ ion count target; maximum injection time 50 ms), followed by top 10 MS2 scans using higher-energy collision dissociation (NCE of 26; 17.5 K resolution, 5 ×10^4^ ion count target; 1.6 m/z isolation window; maximum injection time: 250 ms). Only precursor with charge states between 2-7 were fragmented. Dynamic exclusion was set to 30 s. Raw data were analyzed using the MaxQuant software^74^ (v1.6.3.4). The internal Andromeda search engine was used to search MS2 spectra against a human decoy UniProt database (HUMAN.2019-07) containing forward and reverse sequences. The search included variable modifications of oxidation (M) and N-terminal acetylation, deamidation (N and Q) and fixed modification of carbamidomethyl cysteine. Minimal peptide length was set to 7 amino acids and a maximum of two missed cleavages was allowed. The FDR was set to 1% for peptide and protein identifications. The integrated label-free quantification algorithm was activated. Unique and razor peptides were considered for quantification. Retention times were recalibrated based on the built-in nonlinear time-rescaling algorithm and MS/MS identifications were transferred between LC-MS/MS runs with the “Match between runs” option, in which the maximal retention time window was set to 0.7 min. The resulting text files were used for further analyses using the Perseus software package^75^ (v1.6.2.1). LFQ intensity values were used for quantification. Reverse hits, contaminants and proteins only identified by site were filtered out. Biological replicates for each condition were defined as groups and intensity values were filtered for “minimum value of 3” per group. After log2 transformation missing values were imputed with random noise simulating the detection limit of the mass spectrometer. Differential protein abundance was calculated using two-sample Student´s *t* test and candidate circRNA binder were defined by enrichment (log2 ratio >2) and detection of a minimal number of individual peptides by mass spectrometry (>3 individual peptides).

Expression values from the published mass spectrometry dataset “Tumor Neuroblastoma ALT (Protein) – Westermann – 34 – LFQ - fw2010prot”^35^ consisting of 34 neuroblastoma tumor samples, 12 *MYCN*-amplified and 22 non-amplified, were downloaded from the R2 database (https://hgserver1.amc.nl/cgi-bin/r2/main.cgi) and differential expression calculated.

### Validating absolute circRNA downregulation

DNA was isolated from SK-N-AS cells harboring the inducible *MYCN* expression system after induction with tetracycline for 5 days with NucleoSpin Tissue kit (Macherey-Nagel GmbH & Co. KG, Düren, Germany) according to manufacturer’s instructions. For the calculation of SK-N-AS TR-MYCN cell counts at the DNA level, a specific NRAS SNV c.181C>A mutation was used for an allele-specific quantitative real-time PCR assay (ASQ-PCR), which was designed in accordance with Barz et al.^76^. The NRAS SNV c.181C>A mutation was initially detected using our neuroblastoma-specific hybrid-capture panel sequencing assay^77^. The 3’ end of the allele-specific primer is placed on the SNV mutation to design the ASQ-PCR (sequences listed in Supplementary Data 9), in which the base directly preceding the mutation is unchanged and the third base from the 3’ end is designed with a mismatch (“bridging principle”). The reverse primer was designed to bind downstream of the mutation in an uncritical region (using the hg19 reference genome). The assay also utilized a 20-mer hydrolyzation oligonucleotide probe equipped with a FAM reporter dye (TaqManTM system, 6-carboxyfluorescein amidite) at the 5’ end and a BHQ1 quencher dye (Black Hole Quencher®−1) at the 3’ end. For all quantified samples, the control gene represented a part of the ß-globin (hemoglobin subunit beta) gene locus^78^. Primer and the FAM/BHQ1 probe were manufactured by Eurofins Genomics GmbH. Primer and probe stock solutions (100 pmol/μl) were aliquoted 1:10 with ddH_2_O for further use. PCR-amplified sequences were detected via the FAM tag in real-time. Using a RQ-PCR mixture (3.1 μl ddH2O, 3 μl MgCl2 50 mM (Thermo Fisher Scientific), 2 μl 10× Buffer (Thermo Fisher Scientific), 2 μl dNTPs 2 mM (Bio-Budget Technologies GmbH, Krefeld, Germany), 1 μl forward primer 10 pmol/μl, 1 μl reverse primer 10 pmol/μl, 0.5 μl bovine serum albumin (BSA) 0.20 μM sterile filtered (Carl Roth GmbH+ Co. KG, Karlsruhe, Germany), 0.5 μl FAM-BHQ1 probe 10 pmol/μl, 0.2 μl PlatinumTM Taq DNA polymerase 5 U/μl (Thermo Fisher Scientific)) and the StepOnePlusTM Real-Time PCR System supported by StepOnePlusTM software (Thermo Fischer Scientific), RQ-PCR was performed at 94 °C for 5 min, 94 °C for 8 s, 60 °C (annealing temperature) for 23 s, repeating step 2-3 for 50 times, cool- down to 4 °C. The choice of detection channel was FAM-NFQMGB (non-fluorescent quencher minor groove binder channel). The experimental setup for SK-N-AS TR-MYCN cell count detection and analysis of the ASQ-PCR assays was performed regarding the MRD diagnostic guidelines of van der Velden et al.^79^ and Barz et al.^76^. The total DNA input in one PCR reaction was 670 ng, which corresponds to about 100,000 cells (the amount of DNA in one cell corresponds to 6.7 pg). For quantification of SNVs mutations, the ratio of mutation positive cells to wild-type cells (DNA isolated from leukocytes of healthy donors) in a sample was calculated using standard curve equations.

For absolute normalization of circRNA expression, harvested RNA of 250,000 cells with or without *MYCN*-induction was spiked-in with commercially available External RNA Controls Consortium (ERCC) spike-in (Thermo Fisher) of a defined composition. ERCC were defrosted on ice, freshly diluted 1:40 and 1 µl added to the cells homogenized in Trizol™ (Thermo Fisher Scientific) according to Loven et al.^31^. RNA isolation was performed as described above. The RNA was used for qRT-PCR. Synthesis of cDNA was performed with 500 ng of RNA as described above and cDNA was diluted 1:10. Four circRNAs that were among the most downregulated, as identified by RNA sequencing after 5 days of *MYCN*-induction were profiled by qRT-PCR. Normalization was performed on an average of 2 transcripts of the ERCC spike-in. Please refer to Supplementary Data 9 for primer sequences.

### circARID1A subcellular localization

Circular and linear RNA transcripts from the *ARID1A* gene were visualized in IMR-5 neuroblastoma cells using RNA FISH. Probes targeting the back-splice junction of circARID1A and the 3’UTR of *ARID1A* mRNA were designed (sequences are proprietary to the manufacturer) and generated by ACDBio (Minneapolis, MN, USA) for in situ hybridization using the BaseScope™ Detection Kit v2 – Red standard protocol (ACDBio) according to the manufacturer’s protocol for cultured adherent cells in their technical note for the RNAscope 2.5 Chromogenic Assay. Chamber slides (Merck) were coated with 3.5 μg/cm^2^ CellTak™ Cell and Tissue adhesive (Fisher Scientific, Schwerte, Germany), then seeded with 130,000 cells per 0.7 ×0.7 mm well and incubated 48 h under standard culture conditions. Stained cells were mounted in Vectashield mounting medium with DAPI (Vector Laboratories). Images were acquired on a Nikon Widefield Ti2 EPI fluorescence microscope equipped with a Plan-Apochromat-40x/0.95 DIC air objective with the NIS-Elements Imaging Software (v5.02.00, Nikon, Minato, Japan). The final images are maximum intensity projections and were post-processed with ImageJ (v1.46 m) and Adobe Lightroom (v10.0). The orthogonal validation via cell fractionation used the „*Rapid, efficient and practica*l“ (REAP) method^80^ with IMR-5 cells. Nuclear (*RNU6B*) and cytoplasmic RNA targets were determined by qRT-PCR of subcellular fractions.

### RNA and protein immunoblotting

Western blotting was performed as previously described^81^ using the antibodies listed in in Supplementary Data 9. Protein stability was assessed by treating cells with 30 µg/ml cycloheximide (CHX, Sigma-Aldrich) for 1–24 h and performing western blot afterwards. To assess the effects of circARID1A knockdown on KHSRP protein stability, the knockdown was performed in IMR-5 cells for 4d with subsequent CHX treatment for 24 h starting on day 3. DMSO served as the control treatment. Northern blotting utilized the protocol described by Rybak-Wolf et al.^13^. Briefly, up to 50 µg concentrated total RNA was separated on agarose gels then transferred Hybond N+ membrane (GE Healthcare, Chicago, IL, USA) using the Bio-Rad semi-dry blotting system and UV-crosslinked. Probes spanning the back-splice junction were generated by amplifying the desired sequence (200–300 nt) using Q5 polymerase and a reverse primer that added the T7 promoter sequence, followed by in vitro transcription using T7 RNA polymerase (New England Biolabs) and the DIG RNA labeling mix (Roche) (sequences in Supplementary Data 9). Following purification by precipitation, 50 ng of DIG-labeled probe was used for hybridization. Stringent washing was performed before incubation with anti-digoxigenin-AP Fab fragment (dilution 1:10,000; Roche), and visualization with ready-to-use CDP-Star (Roche). The chemiluminescence signal was detected using the Gel Doc System (Fusion FX, Vilber Lourmat, Collégien, France).

### Chromatin immunoprecipitation (ChIP) DNA sequencing

Publicly available MYCN ChIP-seq data from 3 human *MYCN*-amplified neuroblastoma cell lines (Kelly, SK-N-BE(2)C, NB-1643), 1 non-amplified cell line (NB-69), and from murine medulloblastoma tumorspheres were downloaded from Gene Expression Omnibus (GEO) under Accessions GSE80151^56^ (SK-N-BE(2)C), GSE94782^82^ (NGP, Kelly), GSE138295^82^ (NB-69), and GSE64425^38^ (tumorspheres). We trimmed adapters with BBMap (http://sourceforge.net/projects/bbmap/, v38.58) and aligned the human data to hg19 and the murine data to mm10 using BWA-MEM^83^ (v0.7.15) with default parameters. Duplicate reads were removed with Picard (https://broadinstitute.github.io/picard/, v2.20.4). ChIP-seq mappings were quality controlled with RSC and NSC (*Phantompeakqualtools*^84^ v1.2.1). Reads were extended to 200 bp and filtered by the ENCODE DAC blacklist. Read counts in 10 bp were normalized to CPM using deepTools^85^ (v3.3.0) to prepare ChIP-seq BigWig tracks for visualization in IGV (v2.3.93). We performed peak calling with MACS2^86^ (v2.1.2) using default parameters.

### RNA sequencing

RNA was isolated for sequencing with Trizol™ (Thermo Scientific Fisher) according to the manufacturer’s protocol. RNA purity was analyzed on a Nanodrop 2000 spectrometer (Thermo Scientific Fisher). RNA integrity was assured of being at least 8.0 using a Bioanalyzer 2100 (RNA Nano Chip, Agilent) or TapeStation4200 system (RNA ScreenTape, Agilent). Total RNA sequencing of primary neuroblastoma samples and cell line models with the Illumina short-read sequencing platform was performed as described previously^87^. In short, ribosomal RNA (rRNA) was depleted by enzymatic digestion (based on the protocol described by Adiconis et al.^88^), and ribodepletion was validated by qRT-PCR with primer specific for 18S and 28S rRNA. Depletion of 95–99% rRNA was regularly achieved. Ribodepleted RNA was used to generate total RNA sequencing libraries with the TrueSeq Stranded mRNA kit (Illumina, San Diego, CA, USA) according to manufacturer’s directions. Final libraries were stored short-term at −20 °C until sequencing. Libraries were sequenced on the HiSeq4000 (Illumina) platform with a paired-end read length of 2 × 150 nt and a sequencing depth of 100 million reads at the Sequencing Core Facility of the Max Delbrueck Center for Molecular Medicine (MDC, Berlin, Germany). Poly-A enriched mRNA sequencing libraries were generated with the TrueSeq Stranded mRNA kit (Illumina) by the MDC Core Facility. Libraries were sequenced on the HiSeq4000 (Illumina) platform with a paired-end read length of 2 × 75 nt, generating on average 75 million reads per sample.

Oxford Nanopore long-read sequencing was used to analyze circRNAs in full-length from 6 different neuroblastoma cell lines: IMR-5, LS, SH-EP, SH-SY5Y, Lan-5, SK-N-FI. Sequencing was performed as described before^21^ with a MinION MK1C sequencer using the MinKNOW software (v22.05.8). A detailed and fully referenced protocol was deposited on protocols.io, 10.17504/protocols.io.rm7vzy8r4lx1/v2.

### Processing RNA sequencing data

Sequencing quality was checked using FastQC (v0.11.7). Reads were aligned to GRCh38 using the GENCODE (v30) annotation and the STAR aligner^89^ (v2.7.1a) with the following non-standard parameters: *--sjdbOverhang 300 --twopassMode Basic --chimSegmentMin 12 --chimJunctionOverhangMin 12 --alignSJDBoverhangMin 10 --alignMatesGapMax 200000 --alignIntronMax 200000 --outSAMattributes NH NM MD AS XS nM HI*. Feature counting used featureCounts^90^ (v1.5.1) with the following non-standard parameters when counting at the gene level: *-t exon -g gene_id -C -M --fraction -p -s 2 -O -B -Q 4* and the following non-standard parameters when counting at the exon level: *-t exon -f -J -g gene_id -C -M --fraction -p -s 2 -O -B -Q 4*. Isoform expression in transcripts per million (TPM) units was estimated using kallisto^91^ (v0.44.0) and the following non-standard parameters: *--bootstrap-samples*=*100 --rf-stranded*. Workflows were organized, when possible, with MONSDA (v1.0.0, doi:10.31219/osf.io/jeqgr).

### Whole-genome sequencing and analysis of copy-number variants

Whole-genome sequencing of 64 primary neuroblastoma samples in our cohort was performed as previously described^87^. In short, tumor DNA was provided by the German Neuroblastoma Biobank (Cologne). Sequencing libraries were created by the German Cancer Research Center (DKFZ, Heidelberg, Germany) and sequenced on an Illumina HiSeq X-Ten platform with a paired-end read length of 2 ×150 nt. Read sequences and base quality scores were de-multiplexed and stored in *Fastq* format using Illumina bcl2fastq software (v2.20). Sequence read quality was assessed using FastQC software (v0.11.7). Reads were aligned to the human genome (assembly GRCh38) using BWA-MEM software^83^ (v0.7.10), and duplicate read alignments were removed using samblaster^92^ (v0.1.24). Copy-number alterations were determined as previously described^87^ using Control-FREEC^93^, (v11.0), and compared each tumor sample with the matched peripheral blood control sample from the same patient.

### circRNA discovery

Raw reads were realigned to GRCh38 with BWA-MEM^83^ (v0.7.17-r1188) using the *-T 19* non-standard parameter, and alignments were subsequently processed by CIRI2^94^ (v2.0.6) to predict circRNA candidates. circRNA annotation was also based on GRCh38. The confident set of circRNAs was identified by removing predictions on unplaced GRCh38 assembly contigs and on chrM and chrY. For acceptance, exon-exon annotated back-spliced junctions must belong to the same gene and the acceptor/donor exon boundaries agree up to 1 bp with the GENCODE (v30) annotation. Each circRNA candidate was also required to be either expressed in 25% of all tumors or to have its back-spliced junction supported by at least 20 reads in at least three tumors. The putative sequence of each circRNA in the confident set was constructed by splicing together all annotated exons between the back-splice junction, including the acceptor and donor exons. The longest exon was selected in cases with exon overlaps. The annotation preserved all discrepancies in acceptor/donor exon boundaries. The longest spliced sequence composed from all annotated upstream or all annotated downstream exons was selected as the control sequence for each circRNA. If the sequence was longer than the putative circRNA sequence, it was symmetrically trimmed to equal size from both ends. All circRNAs defined in our cohort were matched with a control sequence of the same length (majority) or longer.

To analyze expression of neuroblastoma-specific circRNAs, publicly available raw total RNA sequencing data were downloaded from the NCBI Gene Expression Omnibus (https://www.ncbi.nlm.nih.gov/geo/) or the Encyclopedia of DNA Elements (ENCODE) consortium (https://www.encodeproject.org). We combined the following datasets from neural tissues to create our “healthy brain tissue” control dataset: (i) postmortem tissue from patients with autism spectrum disorder and matched healthy controls (GSE102741, *n* = 52)^95^, (ii) fetal brain tissue (cerebellum (ENCSR000AEW), occipital lobe (ENCSR000AFD), parietal lobe (ENCSR000AFE), diencephalon (ENCSR000AEX), frontal cortex (ENCSR000AEY), temporal lobe (ENCSR000AFJ), *n* = 12)^96^ and (iii) developing human brain (GSE71315, *n* = 8)^97^. The following datasets from adult and pediatric cancer tissues were combined as the “various tumors” control dataset: (i) high-grade glioma and matched control (GSE62563, *n* = 2)^98^, (ii) pediatric high-grade glioma (GSE95277, *n* = 12)^99^, (iii) pediatric T-cell acute lymphoblastic leukemia (GSE78785, *n* = 13)^100^, (iv) hepatocellular carcinoma with matched controls (GSE77509, *n* = 40)^101^ and (v) mixed cancers (GSE77661, *n* = 19)^102^. Neuroblastoma-specific circRNAs were identified by first identifying the top 500 circRNAs expressed in all tumors by summing circRNA count per million (CPMs) based on CIRI2 counts across isoforms and within each tumor, and then calculating the mean across the tumors. Significant differences were tested between groups using 2 separate Mann–Whitney U tests conducted in tandem (employed to expression CPMs for circRNA isoforms with ≥1 CPM in at least 30% of all tumors) that compared expression CPMs for the same circRNA isoform in our neuroblastoma cohort samples to our combined control datasets from various other cancers or human brain tissue (i.e. healthy normal control). P-values were corrected for the multiple hypothesis testing using the false discovery rate (FDR), and circRNA isoforms that passed both tests with *p*-value < 0.001 were retained. Each of the retained circRNA isoforms was assigned a combined *p*-value, which was the sum of the two p-values associated with the two separate Mann–Whitney U tests.

To analyze DHX9 expression in an independent neuroblastoma tumor cohort, published expression values^34^ from the total RNA-seq dataset “SEQC cohort“ consisting of 498 neuroblastoma tumor samples, 92 MNA and 491 HR_nMNA (5 unclassified tumors were excluded), were downloaded from the R2 database (https://hgserver1.amc.nl/cgi-bin/r2/main.cgi) and differential expression calculated.

To analyze circRNA expression in medulloblastoma tumor samples, published raw total RNA sequencing data were analyzed that were created within the International Cancer Genome Consortium^103^. Data were downloaded from the European Genome-phenome Archive (https://www.ebi.ac.uk/ega) under accession number, EGAD00001003279 (*n* = 39)^39^, after obtaining data access approval.

### Unbiased circRNA and cognate mRNA quantification

We constructed back-spliced junction sequences by splicing the acceptor and donor exons together and trimmed them to a length of 160 nts. When that was not possible the candidate circRNA was removed from this analysis. All unique annotated linear junctions associated with the corresponding gene were kept at their original lengths to form each circRNA, so that the aligner preferentially mapped reads with no back-spliced junction to the exons associated with the linear junctions. If a gene had no linear junctions, then the corresponding circRNAs were removed from the analysis. A reference was then constructed with all unique back-spliced and linear junctions, onto which both mates of the paired-end total RNA sequencing data were separately mapped using BWA-MEM (v0.7.17-r1188) and the non-standard parameters *-T 1 -k 81 -P -S*. Counts over junctions were collected from primary alignments with mapping quality of at least 13 and with proper pair-end mate orientation (i.e. first mate mapped to reverse and second mate mapped to forward sequence). For each back-spliced junction we computed the mean counts over all linear junctions and over linear junctions external to the back-spliced junction. For all subsequent analyses using circRNA and mRNA expression, the corresponding circRNA and mRNA expression was averaged across all isoforms. The circRNA:mRNA ratio was based on expression, and was calculated and shown as an empirical cumulative distribution function (ECDF plot) with the ratio on the x-axis and the probability on the y-axis.

### Differential expression and splicing analysis

All differential expression analyses used the DESeq2 package (v.1.32.0) in R/Bioconductor^104^. circRNA counts were summed at the gene level across isoforms. Most importantly, size factors for the circRNA samples were determined based on gene counts, not circRNA counts, to prevent global changes in circRNA expression from being normalized out. Differential exon coverage was analyzed using the R/Bioconductor^105^ edgeR (v.3.34.1) package. Differential transcript usage was analyzed using SUPPA2^106^ (v.2.3). Human splicing factors and RBPs were identified using the SpliceAid-F database^107^, Molecular Signature Database^42^ (MSigDB, v7.2) and the curated C2 gene sets, “KEGG_Spliceosome (M2044)” and “Reactome_mrna_splicing (M14033)”. Transcriptional MYCN targets in the differential expression analysis were assessed based on published studies defining direct MYCN targets^67,108^. Differential expression of circRNAs in medulloblastoma samples was analyzed with edgeR (v.3.34.1), and considered the above-mentioned set of confident circRNAs that had at least 5 reads in the medulloblastoma dataset. Samples were defined with high *MYCN* or low *MYCN* expression if expression was above the 80th and below the 20th percentile, respectively. The same cutoffs were used to define high or low *DHX9* expression in samples. Significantly differentially expressed circRNAs were defined using a 5% false discovery rate (FDR) cutoff. Significantly different distributions in samples with high or low gene expression was tested with a χ^2^ test.

### Exon and intron sequences used for RBP motif enrichment

To assay global RBP motif enrichments, we constructed the unique sequences of exons and introns by first removing chrM and chrY exons and introns (GENCODE v30 annotation), then filtering out exons or introns <15 nt and >1e6 nt. The corresponding sequences were shuffled, preserving their dinucleotide frequencies, to create control background sequences. Only introns of at least 15 nt (GENCODE v30 annotation) were kept to construct the flanking introns of circRNAs. The flanking introns for each circRNA in the confident set defined above were identified by taking the longest upstream and downstream intron in each case when possible. There were a few cases of circRNAs with either no upstream or no downstream introns available. The corresponding control introns consisted of picking the longest non-overlapping introns of the corresponding gene that also did not overlap with the circRNA or its upstream and downstream introns. In each case, we picked as many control introns as necessary so that their cumulative length was at least as long as the cumulative length of the circRNA flanking introns. No intron splicing was performed in this case, since that would lead to the formation of non-physiological intron-intron junctions that might contribute to the motif counts. Instead, each control intron was assessed separately, as was done for the upstream and downstream circRNA introns. Cumulative intron lengths were used in all comparisons between circRNA introns and controls (i.e. RBP motif density).

### Enrichment of Alu repeats on flanking introns of circRNAs

Introns flanking circular RNAs were divided into upstream and downstream introns. These introns were intersected with detected repeats from RepeatMasker (v4.1.4, www.repeatmasker.org) using bedtools intersect^109^ (v2.30.0). The number of introns intersecting with at least one Alu element was counted and used for comparisons. A Fisher’s exact test was computed for upstream and downstream introns separately, as well as for a combined set of up- and downstream introns.

### Enrichment of RBP motifs on circRNAs and flanking introns

RBP motifs were downloaded from the ATtRACT database^110^. A zero-order Markow model based on the circRNA sequences was used to compute single nucleotide frequencies as the background. Spurious motif entries with position-weight-matrix (PWM) lengths different than their consensus sequence lengths were removed. Motifs of different IDs or consensus sequences that had the same PWMs were merged together into a single entry to avoid overcounting. To look for binding sites based on the reverse-complement of the given motifs, the reverse-complemented motifs and PWMs were converted to the MEME suite^111^ format including the background model nucleotide frequencies. RBP motifs were restricted to 6mers or longer to count RBP motifs in circRNA sequences or assess their enrichment in the flanking introns of circRNAs compared to control intron sequences as background. AME^112^ (MEME suite) was run to estimate the relative enrichment of human RBP 6mer or longer motifs (from the ATtRACT database) in circRNA compared to control sequences. All circRNA sequences were assessed in AME together as a group, with each circRNA sequence being tested separately against the common background. FIMO^113^ (MEME suite) counted separately for each circRNA sequence the RBP motifs that were significantly enriched (*p*-value cutoff <1e−4) compared to a shuffled sequence that preserved the nucleotide frequencies in the whole cohort.

### Proliferative index

We computed the proliferative index (PI) associated with each primary tumor based on the published list of top-correlated genes to the *PCNA* gene^27^. This gene list is incorporated in the ProliferativeIndex CRAN package (v1.0.1)^114^. We used the median of their log2-transformed TPMs as the corresponding PI for each sample, rather than using variance-stabilized counts which are biased by gene length. Neuroblastoma samples were hierarchically clustered based on the Euclidean distance in PI, cutting the tree at the two clusters.

### Assessing variance of expression

The Fano factor is a measure of dispersion. When mRNA is transcribed and degraded under constant rates, then its copy number forms a Poisson distribution with a unity, Fano factor^115^. This means that a unity Fano factor describes the expected dispersion of a constitutively expressed and degraded mRNA. Non-constitutive expression and regulation of an mRNA generally adds noise to the biogenesis resulting in increased dispersion (overdispersion), and tight transcriptional regulation results in underdispersed dynamics where the Fano factor becomes smaller than unity. The Fano factor, defined as the ratio of variance over the mean, was calculated for each circRNA and cognate mRNA based on the circular junction and corresponding external linear junction counts, respectively. They were averaged across isoforms to avoid isoform-specific biases.

### Hierarchical clustering

circRNA expression was used to cluster the 104 neuroblastoma samples in our cohort. Read density was corrected for in each sample by dividing by the sum of reads, before calculating z-scores across samples. Hierarchical clustering utilized the ComplexHeatmap^116^ R package (v2.6.2). The distance-based methods, ‘manhattan’ (for columns) and ‘canberra’ (for rows) were applied. The identified RNA-binding proteins were clustered based on the correlation of their expression with the expression of identified circRNAs. Spearman correlation coefficients were calculated and hierarchically clustered by the Euclidean distance of the coefficients. Counts were normalized for cluster size by dividing them with the product of the corresponding number of RBPs and circRNAs for each cluster.

### Enrichment of Gene-Ontology terms and gene signatures

Overrepresentation of Gene Ontology (GO) terms (from the category of biological processes) in sequencing datasets was calculated using annotated GO terms with the PANTHER^117^ tool (v14) using Fisher’s exact test. All expressed genes (base mean of at least 10) were used as a background, and results were plotted using ggplot2 (v3.0.0). Similarly, overrepresentation of gene signatures annotated in the Molecular Signature Database^42^ in the collection C2 Curated Gene Sets among the differentially expressed genes was investigated.

### Data visualization

A Circos plot was generated with the R package circlize^118^ (v0.4.5) to visualize the location of genes producing circRNAs and mRNAs on the genome-wide level. The ratio of circRNA and mRNA per gene was calculated. Genes having a ratio of >1.2 were considered *circRNA-productive genes*. The location of *circRNA-productive genes* in function of genomic areas with copy-number aberrations was analyzed and is shown in the Circos plot. An Oncoplot was generated with the R package ComplexHeatmap^116^ (v1.10.2) showing different clinical and molecular features of the analyzed neuroblastomas. In order to visualize the overlap of two datasets, we drew pairwise Venn diagrams using the R package VennDiagram^119^ (v1.6.20).

### Statistical analysis

Statistics for bioinformatics analysis is mentioned in the respective methods paragraph. Comparisons of differential expression were tested with rank sum-based tests. Enrichment or overlap among datasets were analyzed with Fisher’s exact test. Results generated by qRT-PCR were analyzed using the 2^ΔΔCT^ method (when comparing to the average of control samples, as for knockdowns) or the 2^ΔCT^-method (when comparing to the average of untreated conditions in RNaseR and actinomycin D treatments). If not stated otherwise, all in vitro experiments were performed in 3 independent biological replicates. Statistical significance among or between treatment groups in in vitro experiments was determined by one-way (to analyze 1 parameter) or two-way ANOVA (to analyze more than 1 parameter) for more than 2 groups, or unpaired t-tests for 2 groups, using Microsoft Excel 2016 or GraphPad Prism 9 (GraphPad Software). For statistical analysis of SK-N-AS proliferation, Richards models were fitted to the growth curves and initial proliferation rates were calculated using the ‘growthrates’ R package (https://CRAN.R-project.org/package=growthrates, v0.8.2). The low proliferation of cells with knockdown and ASO treatment made fitting growth models inconvenient. Instead, area under the curve (AUC) was calculated using the trapezoid method in the ‘DescTools’ R package (https://cran.r-project.org/package=DescTools, v0.99.40). Wilcoxon-rank tests were used to test significance in pairwise comparisons. For all tests, a *p*-value < 0.05 was considered as statistically significant. Graphs show the mean and error bars representing standard deviation. In box plots the center line represents the median, boxes indicate the interquartile range, the whiskers show the range. *p < 0.05, **p < 0.01, ***p < 0.001, ****p < 0.0001 and ns, not significant.

### Reporting summary

Further information on research design is available in the Nature Portfolio Reporting Summary linked to this article.

## Supplementary information

Supplementary Information

Description of Additional Supplementary Files

Supplementary Data 1

Supplementary Data 2

Supplementary Data 3

Supplementary Data 4

Supplementary Data 5

Supplementary Data 6

Supplementary Data 7

Supplementary Data 8

Supplementary Data 9

Reporting Summary

## Data Availability

The RNA sequencing and ChIP sequencing data used in this study are publicly available in the NCBI Gene Expression Omnibus under accession codes GSE102741^95^, GSE71315^97^, GSE62563^98^, GSE95277^99^, GSE78785^100^, GSE77509^101^, GSE77661^102^, GSE80151^56^, GSE94782^82^, GSE138295^82^, GSE64425^38^, from the ENCODE consortium under accession numbers ENCSR000AEW, ENCSR000AFD, ENCSR000AFE, ENCSR000AEX, ENCSR000AEY, ENCSR000AFJ^96^ and from the European Genome-phenome Archive under accession number EGAD00001003279^39^ after approval by the data access committee. The genome references hg38 (https://ftp.ensembl.org/pub/release-109/fasta/homo_sapiens/dna/Homo_sapiens.GRCh38.dna.alt.fa.gz), hg19 (https://ftp.ensembl.org/pub/grch37/current/fasta/homo_sapiens/dna/Homo_sapiens.GRCh37.dna.alt.fa.gz) and mm10 (ftp://ftp.ensembl.org/pub/release-102/fasta/mus_musculus/dna/) were downloaded from the ensemble website (https://ensemblgenomes.org) and the transcriptome annotation Gencode v30 from the gencode website (https://www.gencodegenes.org/human/release_30.html). The mass spectrometry data generated in this study have been publicly deposited in the PRIDE repository under accession code PXD026053. The RNA sequencing and Whole Genome Sequencing data of neuroblastoma patients are deposited in the European Genome-phenome Archive under accession codes EGAS00001004022, EGAS00001005604. This dataset from neuroblastoma patients is available under restricted access due to data privacy laws. Access to the EGA archive dataset is obtained by formal application to the Data Access Committee (DAC, https://ega-archive.org/dacs/EGAC00001002310) at johannes.schulte@charite.de. The DAC will honor legitimate requests for sequencing data from researchers as necessary for conducting methodologically sound research for precise projects. The DAC requires users/applicants to sign a Data Access Agreement (DAA), which details the terms and conditions of use for each dataset, for example to use the data only for the project that was applied for, and to not share the data with other parties. The DAC will respond to requests within 2 weeks and provide access to the data within 4 weeks. The data will be made available for 12 months, once the DAA is signed by both sides. Further RNA sequencing and Nanopore data have been publicly deposited at the NCBI Gene Expression Omnibus under accession numbers GSE174571, GSE174572, GSE174708, GSE181561, GSE223105. The remaining data are available within the Article, Supplementary Information or Source Data file. Further information and requests for resources and reagents should be directed to and will be fulfilled by the Lead Contact, Johannes H. Schulte, johannes.schulte@charite.de. Source data are provided with this paper.

## Source data

Source Data

## Code availability

Scripts and code used for sequencing data analysis in this study are fully referenced in the methods section. We further deposited codes and scripts to reproduce our analysis on Github (https://github.com) under the following link: 10.5281/zenodo.7817607^120^.
