# Supplementary material for: Defining the landscape of circular RNAs in neuroblastoma unveils a global suppressive function of MYCN
Source: Nat Commun. 2023 Jul 4;14:3936. doi: 10.1038/s41467-023-38747-4 (PMC10319804; doi:10.1038/s41467-023-38747-4)
Supplement: Supplementary file 1 — Supplementary Information [file 41467_2023_38747_MOESM1_ESM.pdf]

Supplementary information for

Defining the landscape of circular RNAs in neuroblastoma unveils a global suppressive function of MYCN

Steffen Fuchs*, Clara Danßmann, Filippos Klironomos, Annika Winkler, Jörg Fallmann, Louisa-Marie Kruetzfeldt, Annabell Szymansky, Julian Naderi, Stephan H. Bernhart, Laura Grunewald, Konstantin Helmsauer, Elias Rodriguez-Fos, Marieluise Kirchner, Philipp Mertins, Kathy Astrahantseff, Christin Suenkel, Joern Toedling, Fabienne Meggetto, Marc Remke, Peter F. Stadler, Patrick Hundsdoerfer, Hedwig E. Deubzer, Annette Künkele, Peter Lang, Jörg Fuchs, Anton G. Henssen, Angelika Eggert, Nikolaus Rajewsky, Falk Hertwig, Johannes H. Schulte*

* Correspondence: johannes.schulte@med.uni-tuebingen.de, steffen.fuchs@charite.de.

Fig. S1

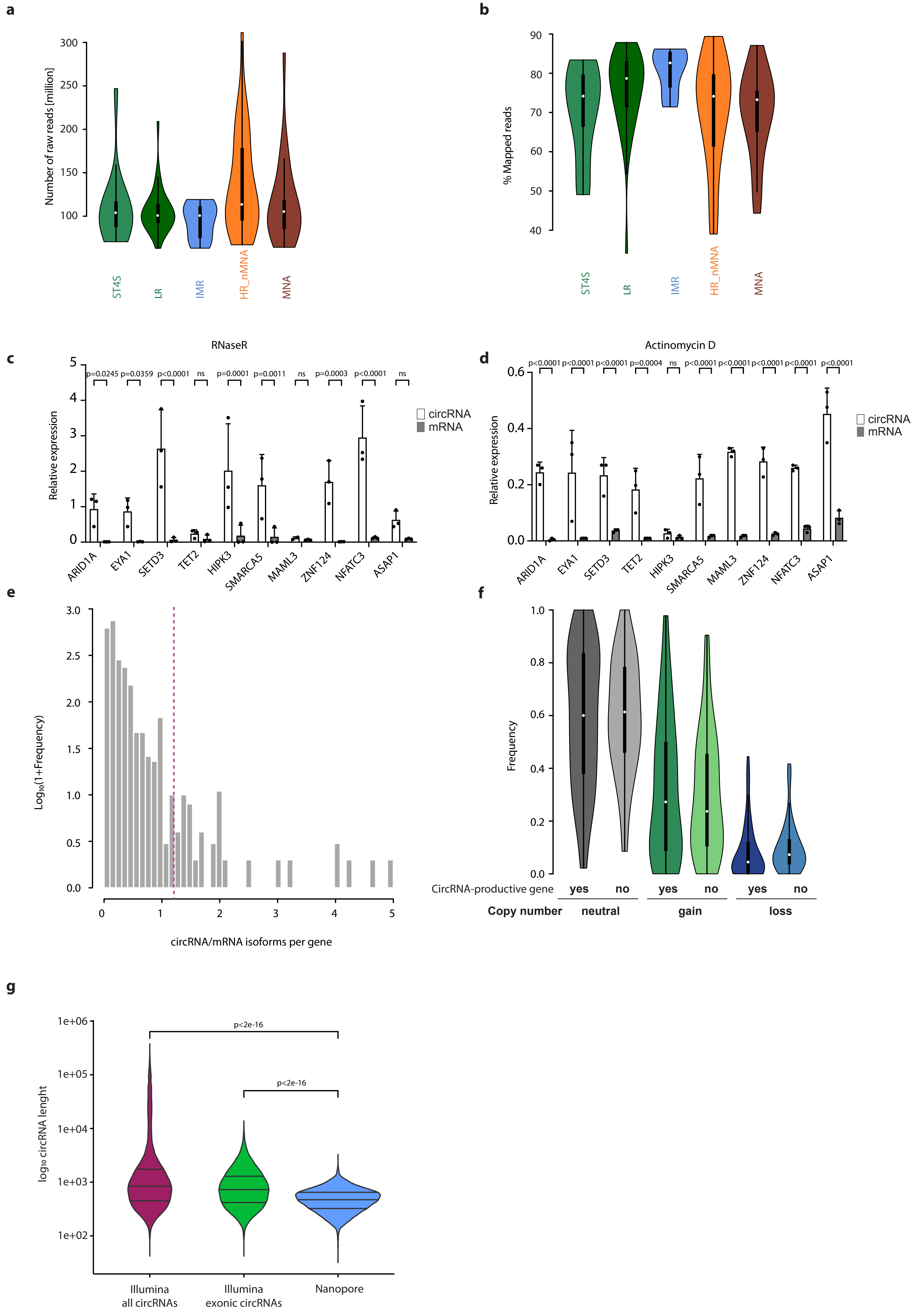

a
Number of raw reads [million]
300
250
200
150
100
ST4S
LR
IMR
HR_nMNA
MNA
b
% Mapped reads
90
80
70
60
50
40
ST4S
LR
IMR
HR_nMNA
MNA
c
RNaseR
Relative expression
p=0.0245
p=0.0359
p<0.0001
ns
p=0.0001
p=0.0011
ns
p=0.0003
p<0.0001
ns
circRNA
mRNA
ARID1A
EYA1
SETD3
TET2
HIPK3
SMARCA5
MAML3
ZNF124
NFATC3
ASAP1
d
Actinomycin D
Relative expression
p<0.0001
p<0.0001
p<0.0001
p=0.0004
ns
p<0.0001
p<0.0001
p<0.0001
p<0.0001
p<0.0001
circRNA
mRNA
ARID1A
EYA1
SETD3
TET2
HIPK3
SMARCA5
MAML3
ZNF124
NFATC3
ASAP1
e
Log10(1+Frequency)
circRNA/mRNA isoforms per gene
f
Frequency
CircRNA-productive gene
yes
no
yes
no
yes
no
Copy number
neutral
gain
loss
g
log10 circRNA lenght
p<2e-16
p<2e-16
1e+06
1e+05
1e+04
1e+03
1e+02
Illumina
all circRNAs
Illumina
exonic circRNAs
Nanopore

Figure S1| circRNAs detected by RNA sequencing are validated and their frequency is not dependent on chromosomal gain or loss.

a-b, Primary neuroblastoma tumor samples (n=104 biologically independent samples) have been analyzed by total RNA sequencing. Violin plots showing sequencing statistics. **a,** Generated raw reads per risk-group. **b**, Mappable reads per risk-group.

c-d, Validation of 10 circRNAs by qRT-PCR in comparison to the cognate mRNA in IMR-5/75 cells (n=3 biologically independent experiments, Data are presented as mean ± SD, Two-way ANOVA test). **c**, Assessment of circularity after RNaseR treatment, **d**, Evaluation of transcript stability after actinomycin D treatment.

e, Global distribution of the ratios of circRNA/mRNA isoforms produced by genes as determined in our neuroblastoma tumor cohort (n=104 biologically independent samples). The cutoff for circRNA-producing genes is marked as used for the Circos plot in **Figure 1d**.

f, The frequency of circRNAs derived from *circRNA-productive genes* associated with, or independent of copy-number neutral, gain or loss regions as determined by whole-genome sequencing of matched neuroblastoma tumors (n=64 biologically independent samples) is shown. Data are presented as a violin plot.

g, The length of circRNAs was estimated based on short-read Illumina RNA sequencing data of neuroblastoma tumor samples (n=104 biologically independent samples) for all circRNAs, only exonic circRNAs, and in comparison to Oxford Nanopore long-read sequencing data of 6 neuroblastoma cell lines (n=6 biologically independent experiments). Data are presented as a violin plot. Violin plots use normal optimal smoothing. The median (white dot), quartiles (box), and 1.5-fold interquartile range (whiskers) are displayed. Wilcoxon Rank Sum test, two sided. Source data are provided as a Source Data file.

Fig. S2

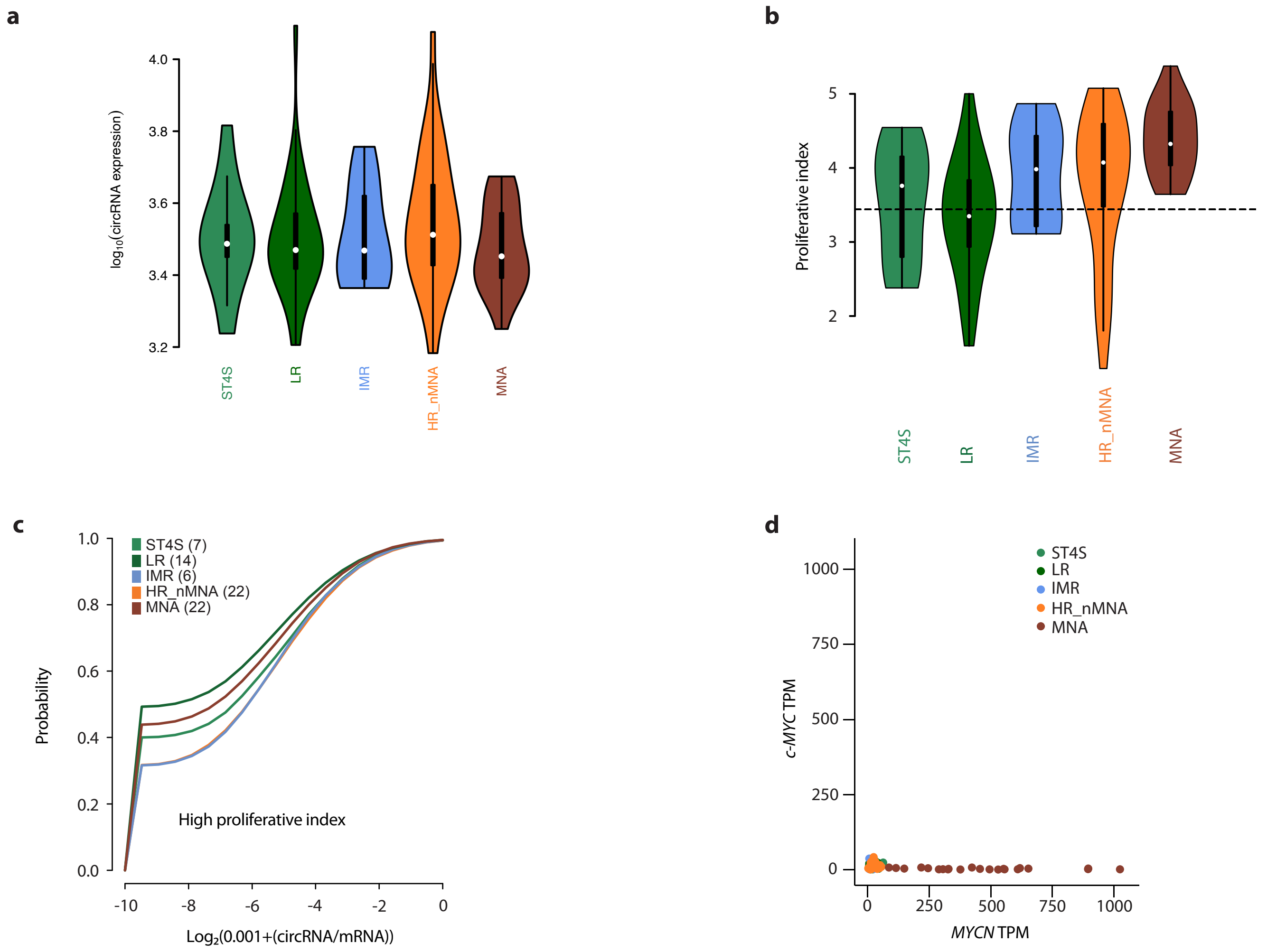
a
log10(circRNA expression)
4.0
3.8
3.6
3.4
3.2
ST4S
LR
IMR
HR_nMNA
MNA
b
Proliferative index
5
4
3
2
ST4S
LR
IMR
HR_nMNA
MNA
c
Probability
1.0
0.8
0.6
0.4
0.2
0.0
ST4S (7)
LR (14)
IMR (6)
HR_nMNA (22)
MNA (22)
High proliferative index
-10
-8
-6
-4
-2
0
Log2(0.001+(circRNA/mRNA))
d
c-MYC TPM
1000
750
500
250
0
ST4S
LR
IMR
HR_nMNA
MNA
0
250
500
750
1000
MYCN TPM

Figure S2| circRNA expression is not biased by proliferation in MYCN-amplified neuroblastoma.

a, Expression of circRNAs, measured as a ratio of circRNA over cognate mRNA expression, per risk group in neuroblastoma tumor samples (n=104 biologically independent samples). Data are presented as a violin plot.

b, Distribution of the proliferative index per risk group of neuroblastoma tumor samples (n=104 biologically independent samples). The threshold for tumors considered to have a high proliferative index used for **c** is marked by a dashed line. ST4S, stage 4S; LR, low risk; IMR, intermediate risk; HR_nMNA, high-risk non *MYCN*-amplified; MNA, *MYCN*-amplified. Data are presented as a violin plot.

c, Expression of circRNAs was analyzed similar to **Figure 3b** as a ratio of circRNA over mRNA, but using only the tumors in the high-proliferative index group. Number of tumors with high proliferative index per risk group is shown in parenthesis.

d, Expression of *c-MYC* and *MYCN* is shown in our neuroblastoma tumor samples (n=104 biologically independent samples). Violin plots use normal optimal smoothing. The median (white dot), quartiles (box), and 1.5-fold interquartile range (whiskers) are displayed. Source data are provided as a Source Data file.

Fig. S3

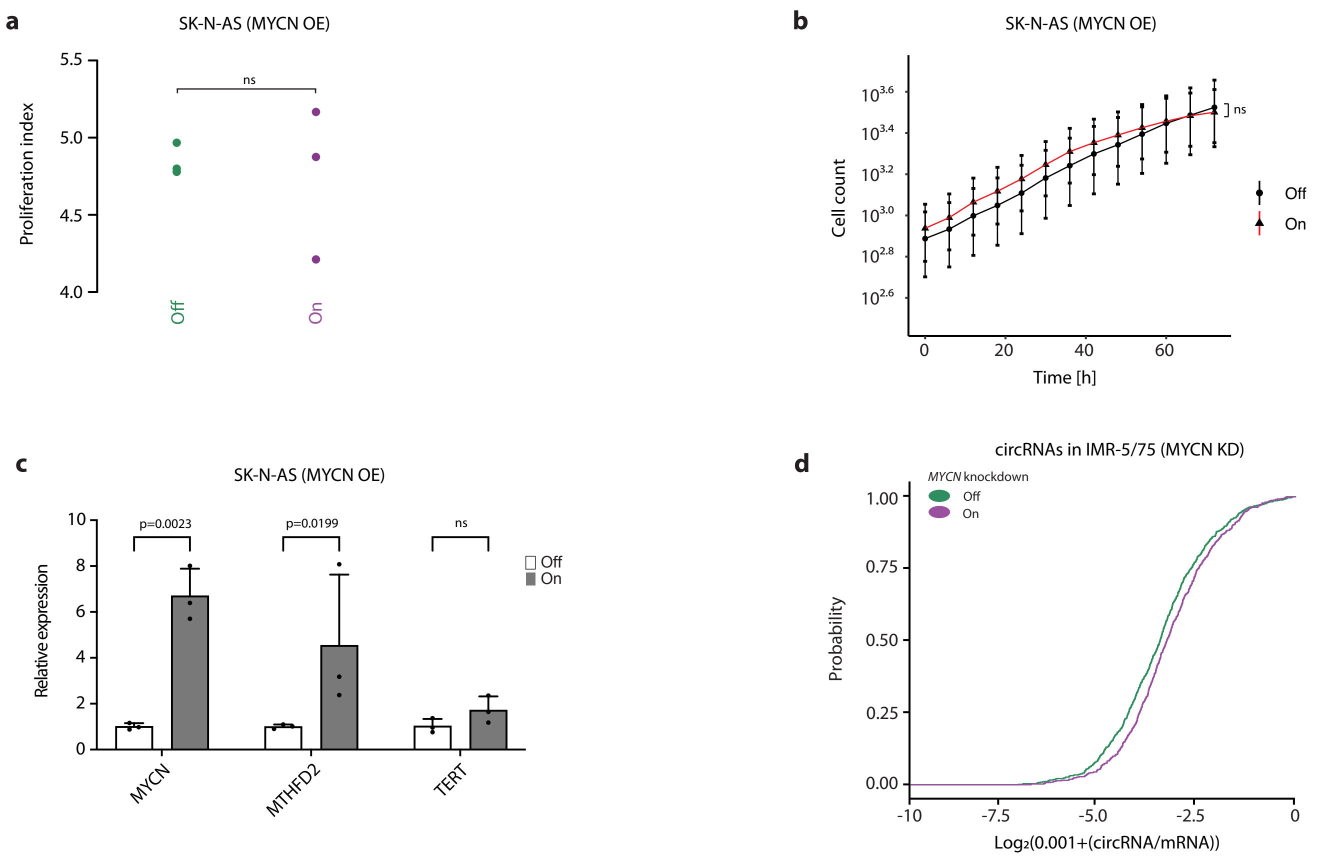

a
SK-N-AS (MYCN OE)
Proliferation index
5.5
5.0
4.5
4.0
ns
Off
On
b
SK-N-AS (MYCN OE)
Cell count
$10^{3.6}$
$10^{3.4}$
$10^{3.2}$
$10^{3.0}$
$10^{2.8}$
$10^{2.6}$
0
20
40
60
Time [h]
ns
Off
On
c
SK-N-AS (MYCN OE)
Relative expression
10
8
6
4
2
0
p=0.0023
p=0.0199
ns
Off
On
MYCN
MTHFD2
TERT
d
circRNAs in IMR-5/75 (MYCN KD)
MYCN knockdown
Off
On
Probability
1.00
0.75
0.50
0.25
0.00
-10
-7.5
-5.0
-2.5
0
Log2(0.001+(circRNA/mRNA))

Figure S3| Validation of the inducible *MYCN*-expression system.

a, Proliferative index was calculated based on RNA-sequencing data of the inducible *MYCN* expression system in SK-N-AS cells (**Figure 3c**) as in **Supplementary Fig. 2** and non-induced cells (off) were compared with induced cells (on) (n=3 biologically independent experiments, Unpaired two-sided Mann Whitney-U test).

b, Real-time proliferation analysis after 48 h of induction of *MYCN* expression in SK-N-AS inducible cells (n=3 biologically independent experiments, Data are presented as median ± range, Unpaired two-sided Mann Whitney-U test).

c, Assessment of the expression of *MYCN* and the direct published targets *MTHFD2* and *TERT* after 120 h induction of SK-N-AS cells by qRT-PCR (n=3 biologically independent experiments, Data are presented as mean ± SD, Two-way ANOVA test).

d, MYCN knockdown was induced for 120h in IMR-5/75 cells harboring an inducible shRNA to target MYCN. Total RNA sequencing was performed and the ratio of circRNA to linear RNAs was calculated Unpaired two-sided Mann Whitney-U test). The knockdown was confirmed by western blot (**Fig. 4f**). Source data are provided as a Source Data file.

Fig. S4

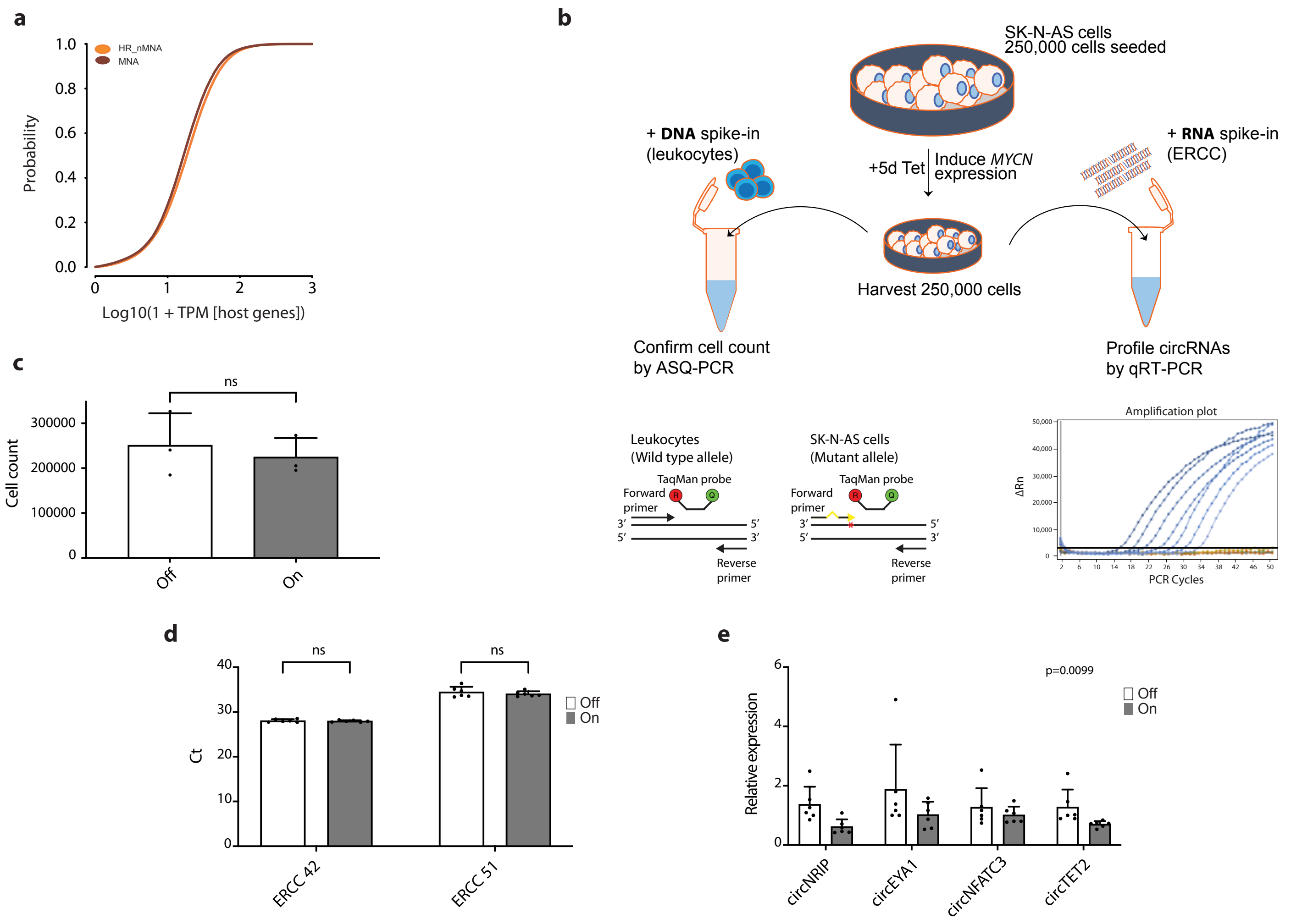

a
HR_nMNA
MNA
Probability
1.0
0.8
0.6
0.4
0.2
0.0
0
1
2
3
Log10(1 + TPM [host genes])
b
SK-N-AS cells
250,000 cells seeded
+ DNA spike-in
(leukocytes)
+5d Tet
Induce MYCN expression
+ RNA spike-in
(ERCC)
Harvest 250,000 cells
Confirm cell count
by ASQ-PCR
Profile circRNAs
by qRT-PCR
Leukocytes
(Wild type allele)
TaqMan probe
Forward primer
Reverse primer
SK-N-AS cells
(Mutant allele)
Amplification plot
ΔRn
PCR Cycles
c
ns
Cell count
300000
200000
100000
0
Off
On
d
ns
ns
Ct
40
30
20
10
0
Off
On
ERCC 42
ERCC 51
e
p=0.0099
Off
On
Relative expression
6
4
2
0
circNRIP
circEYA1
circNFATC3
circTET2

Figure S4| MYCN induction leads to an absolute downregulation of circRNAs.

a, Distribution of the expression (as transcripts per million, TPMs) of host genes that produce circRNAs in *MYCN*-amplified (MNA, n=22 biologically independent samples) and high-risk non *MYCN*-amplified (HR_nMNA, n=29 biologically independent samples) neuroblastoma tumor samples. Mann-Whitney U test, two-sided, p<1e-16.

b, Scheme showing the experimental workflow to validate an absolute downregulation of circRNAs in the inducible *MYCN* expression system based on SK-N-AS cells. Tet, tetracyclin; ERCC, External RNA Controls Consortium; ASQ-PCR, allele-specific RQ-PCR.

c, Assessment of cell count of non-induced (off) vs. induced (on) SK-N-AS cells by ASQ-PCR (n=3 biologically independent experiments, Data are presented as mean ± SD, unpaired two-sided Student's t-test).

d, Detection of ERCC spike-in transcript 42 and 51 by qRT-PCR in non-induced (off) vs. induced (on) SK-N-AS cells (n=6 biologically independent experiments, Data are presented as mean ± SD, Two-way ANOVA test).

e, Expression of 4 circRNAs determined by qRT-PCR after *MYCN* induction normalized to an average of ERCC spike-in transcript 42 and 51 (n=6 biologically independent experiments, Data are presented as mean ± SD, Two-way ANOVA test). Source data are provided as a Source Data file.

Fig. S5

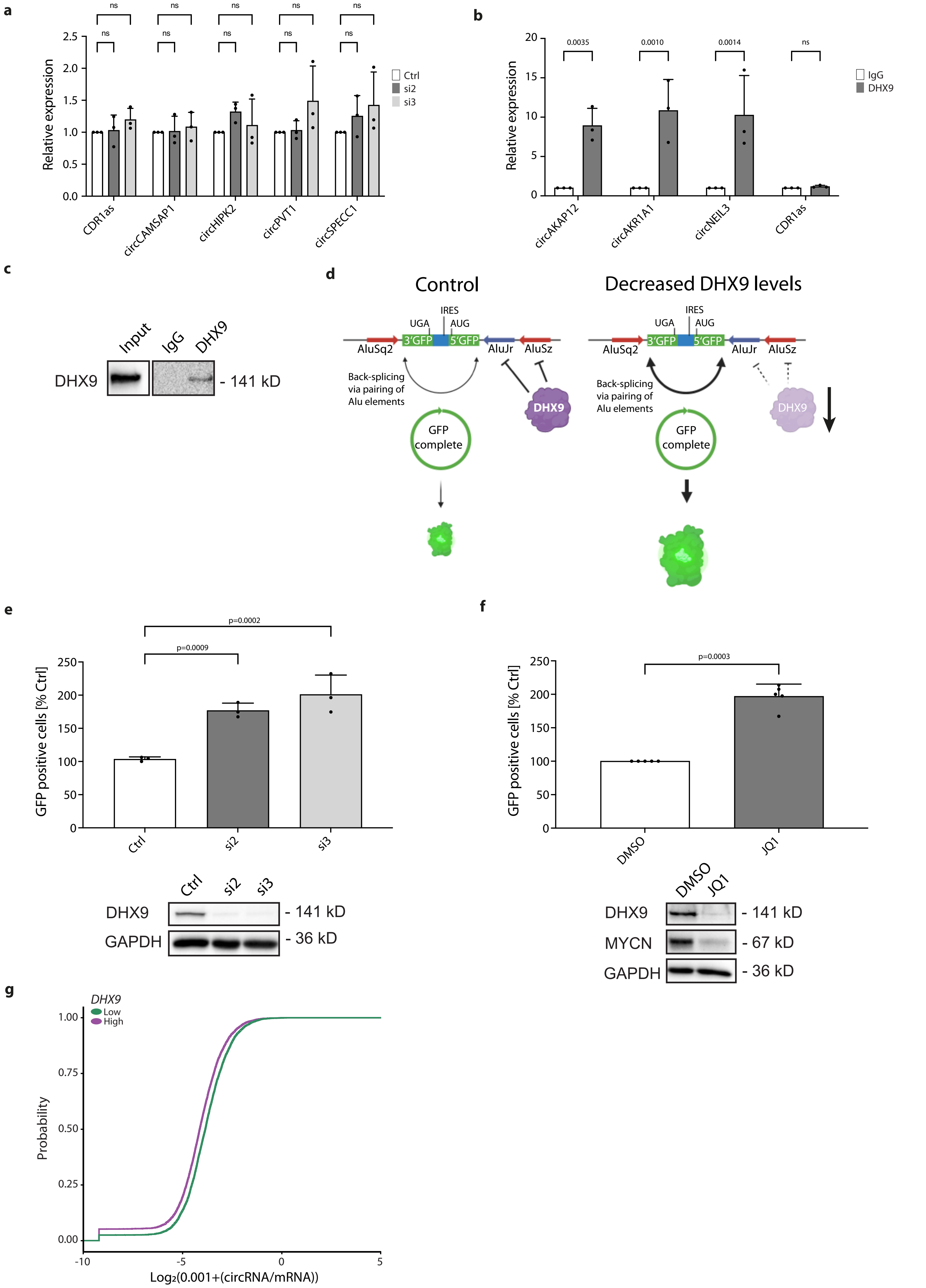
a
Relative expression
Ctrl
si2
si3
ns
CDR1as
circCAMSAP1
circHIPK2
circPVT1
circSPECC1
b
Relative expression
IgG
DHX9
0.0035
0.0010
0.0014
ns
circAKAP12
circAKR1A1
circNEIL3
CDR1as
c
Input
IgG
DHX9
DHX9
- 141 kD
d
Control
Decreased DHX9 levels
IRES
UGA
AUG
3'GFP
5'GFP
AluSq2
AluJr
AluSz
Back-splicing via pairing of Alu elements
DHX9
GFP complete
e
GFP positive cells [% Ctrl]
p=0.0002
p=0.0009
Ctrl
si2
si3
DHX9
GAPDH
- 141 kD
- 36 kD
f
GFP positive cells [% Ctrl]
p=0.0003
DMSO
JQ1
DHX9
MYCN
GAPDH
- 141 kD
- 67 kD
- 36 kD
g
DHX9
Low
High
Probability
Log2(0.001+(circRNA/mRNA))

Figure S5| DHX9 regulates circRNAs enriched in Alu-repeats in their flanking introns.

a, DHX9 knockdown was performed in IMR-5 neuroblastoma cells with 2 different siRNAs and circRNAs that were reported not to be regulated by DHX9 were profiled by qRT-PCR (n=3 biologically independent experiments, Data are presented as mean ± SD, Two-way ANOVA test, related to **Fig. 4i**).

b, RNA immunoprecipitation (RIP) was performed for DHX9 in IMR-5 cells and enrichment of circRNAs was analyzed by qRT-PCR with primer pairs targeting flanking introns (n=3 biologically independent experiments, Data are presented as mean ± SD, Two-way ANOVA test).

c, Specificity of RIP in **b** was validated by western blot. Anti-IgG antibody served as control (n=3 biologically independent experiments).

d, Scheme showing the reporter assay to demonstrate the effect of different DHX9 levels on RNA circularization. A non-functional split *GFP* is flanked by introns from the *ZKSCAN1* gene that harbor several Alu elements. This helps to circularize the *GFP* and render it functional. DHX9 inhibits this process by binding to the Alu elements. DHX9 downregulation fosters circularization and leads to more functional *GFP*. AUG, start codon; UGA, stop codon; IRES, internal ribosomal entry site; AluSq2, AluJr, AluSz, different Alu repeat elements. Figure created with BioRender.com.

e, DHX9 knockdown was performed in SH-EP cells harboring the reporter system and GFP positive cells were measured by flow cytometry in comparison to the control knockdown. Knockdown was validated by western blot (n=3 biologically independent experiments, Data are presented as mean ± SD, One-way ANOVA test).

f, MYCN was inhibited with the BET bromodomain inhibitor JQ1 in IMR-5 cells and GFP positive cells were measured by flow cytometry in comparison to the control treatment with DMSO (n=5 biologically independent experiments, Data are presented as mean ± SD, Two-sided paired Student's t-test). Downregulation of MYCN and DHX9 was validated by western blot.

g, Neuroblastoma tumor samples were clustered in high and low DHX9 expressers and the effect on circRNA/linRNA expression ratios was analyzed (n=104 biologically independent samples, Wilcoxon Rank Sum test, two-sided, p<2e-16). Source data are provided as a Source Data file.

Fig. S6

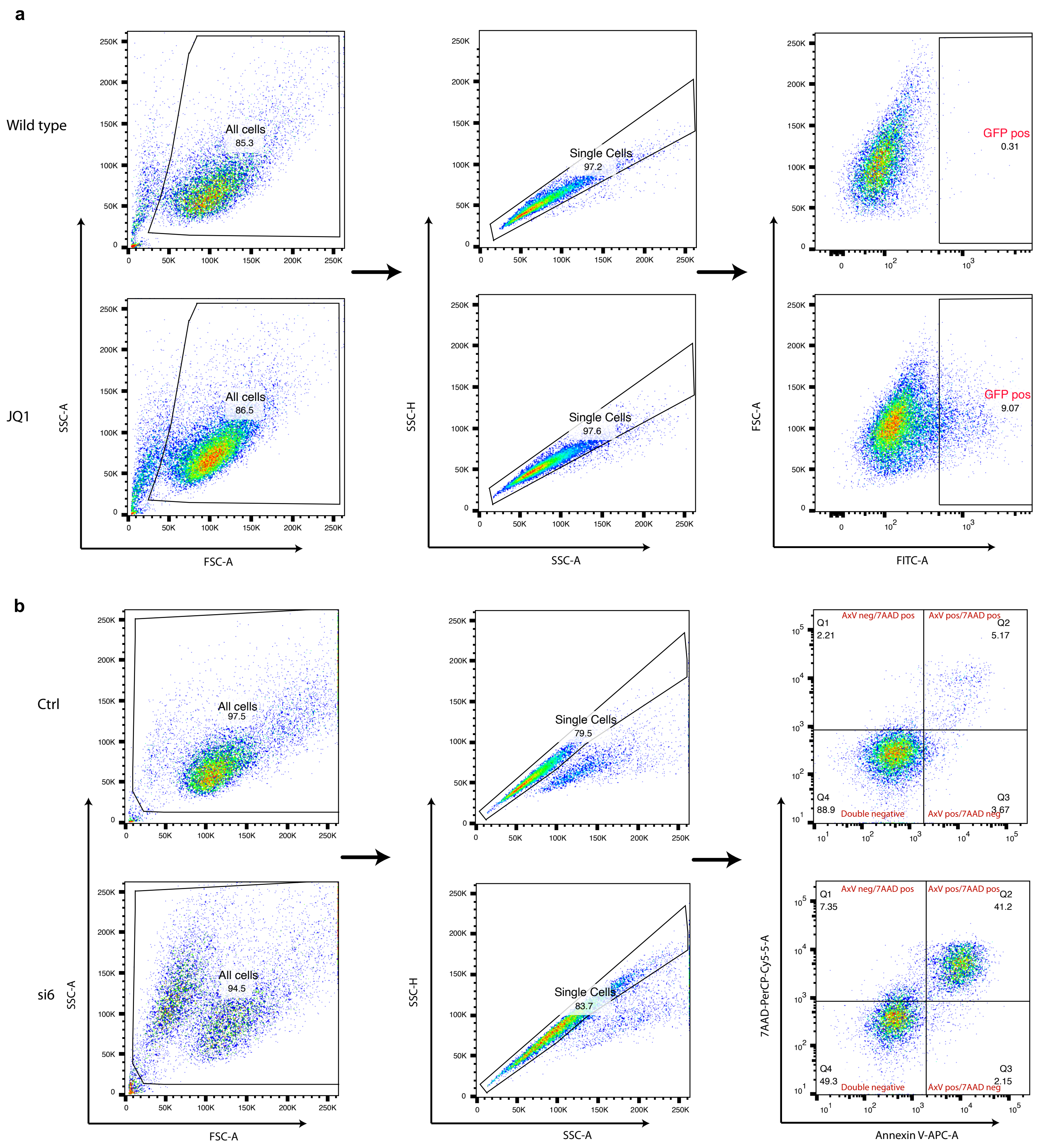
a
Wild type
JQ1
All cells
85.3
All cells
86.5
Single Cells
97.2
Single Cells
97.6
GFP pos
0.31
GFP pos
9.07
FSC-A
SSC-A
SSC-H
FITC-A
b
Ctrl
si6
All cells
97.5
All cells
94.5
Single Cells
79.5
Single Cells
83.7
AxV neg/7AAD pos
AxV pos/7AAD pos
Double negative
AxV pos/7AAD neg
Q1
2.21
Q2
5.17
Q4
88.9
Q3
3.67
Q1
7.35
Q2
41.2
Q4
49.3
Q3
2.15
7AAD-PerCP-Cy5-5-A
Annexin V-APC-A

Figure S6| Representative flow cytometry gating used in this study.

a, Gating strategy for the detection of GFP positive cells in wild type cells and cells harboring the ZKSCAN1 plasmid after JQ1 treatment in the DHX9 reporter assay.

b, Gating strategy for the detection of apoptotic cells as indicated by Annexin V in control (Ctrl) and knockdown cells (si6).

Fig. S7

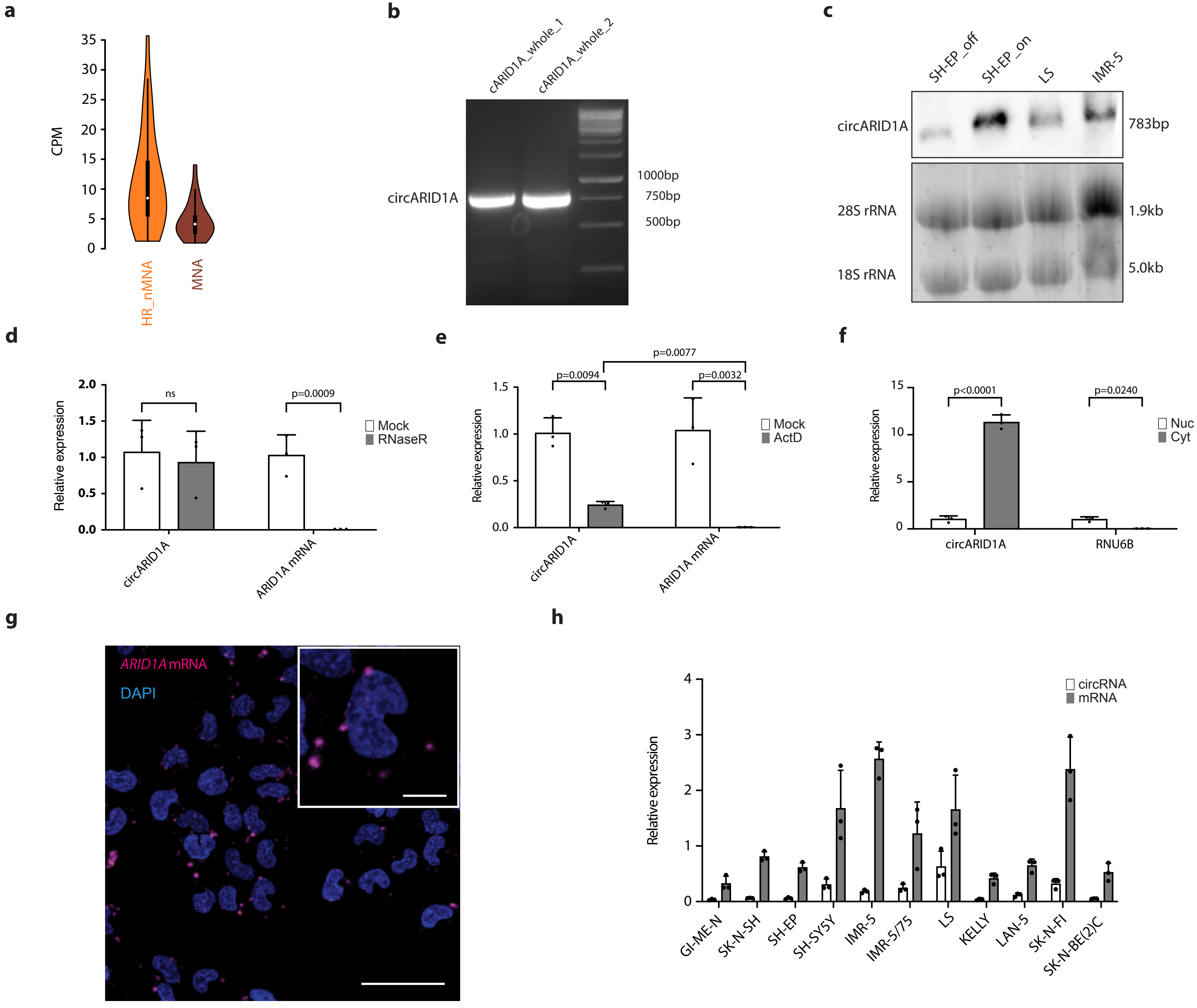
a
CPM
35
30
25
20
15
10
5
0
HR_nMNA
MNA
b
cARID1A_whole_1
cARID1A_whole_2
circARID1A
1000bp
750bp
500bp
c
SH-EP_off
SH-EP_on
LS
IMR-5
circARID1A
783bp
28S rRNA
1.9kb
18S rRNA
5.0kb
d
Relative expression
2.0
1.5
1.0
0.5
0.0
ns
p=0.0009
Mock
RNaseR
circARID1A
ARID1A mRNA
e
Relative expression
1.5
1.0
0.5
0.0
p=0.0094
p=0.0077
p=0.0032
Mock
ActD
circARID1A
ARID1A mRNA
f
Relative expression
15
10
5
0
p<0.0001
p=0.0240
Nuc
Cyt
circARID1A
RNU6B
g
ARID1A mRNA
DAPI
h
Relative expression
4
3
2
1
0
circRNA
mRNA
GI-ME-N
SK-N-SH
SH-EP
SH-SY5Y
IMR-5
IMR-5/75
LS
KELLY
LAN-5
SK-N-FI
SK-N-BE(2)C

Figure S7| circARID1A was validated in vitro in neuroblastoma cells.

a, Expression of circARID1A in *MYCN*-amplified (MNA, n=22 biologically independent samples) and high-risk non MYCN-amplified (HR_nMNA, n=29) neuroblastoma tumors as CPM (counts per million). Data are presented as a violin plot. Violin plots use normal optimal smoothing. The median (white dot), quartiles (box), and 1.5-fold interquartile range (whiskers) are displayed.

b, The whole 783 nt circARID1A was entirely amplified by RT-PCR with 2 different primer pairs and detected by agarose gel electrophoresis (n=1).

c, Direct detection of circARID1A by northern blotting in LS and IMR-5 cells and in SH-EP cells that harbor an inducible expression system for circARID1A (as in **Supplementary Fig. 8**). The input was controlled using an RNA agarose gel analysis. Ribosomal RNA bands are indicated (n=2 biologically independent experiments). Off, non-induced; on, induced.

d, circARID1A und *ARID1A* mRNA expression after exonuclease (RNaseR) treatment of IMR-5/75 cells (n=3 biologically independent experiments, Data are presented as mean ± SD, two-way ANOVA test, as in Supplementary Fig. 1).

e, circARID1A und *ARID1A* mRNA expression after actinomycin D (ActD) treatment of IMR-5/75 cells (n=3 biologically independent experiments, Data are presented as mean ± SD, two-way ANOVA test, two-sided paired Student's t-test for comparison of circRNA with mRNA, as in Supplementary Fig. 1).

f, Detection of circARID1A and RNU6B (nuclear marker) after cell fractionation in IMR-5 cells by qRT-PCR (n=3 biologically independent experiments, Data are presented as mean ± SD, two-way ANOVA test); Nuc, nucleus; cyt, cytoplasm.

g, Direct detection of *ARID1A* mRNA (red color) by RNA-FISH in IMR-5 cells. Nuclei are stained with DAPI (blue color). Insert was digitally magnified. Scale: large image 50 µm, small image 10 µm (n=2 biologically independent experiments).

h, Expression of circARID1A and *ARID1A* mRNA across 11 neuroblastoma cell lines by qRT-PCR (n=3 biologically independent experiments, Data are presented as mean ± SD). Source data are provided as a Source Data file.

Fig. S8

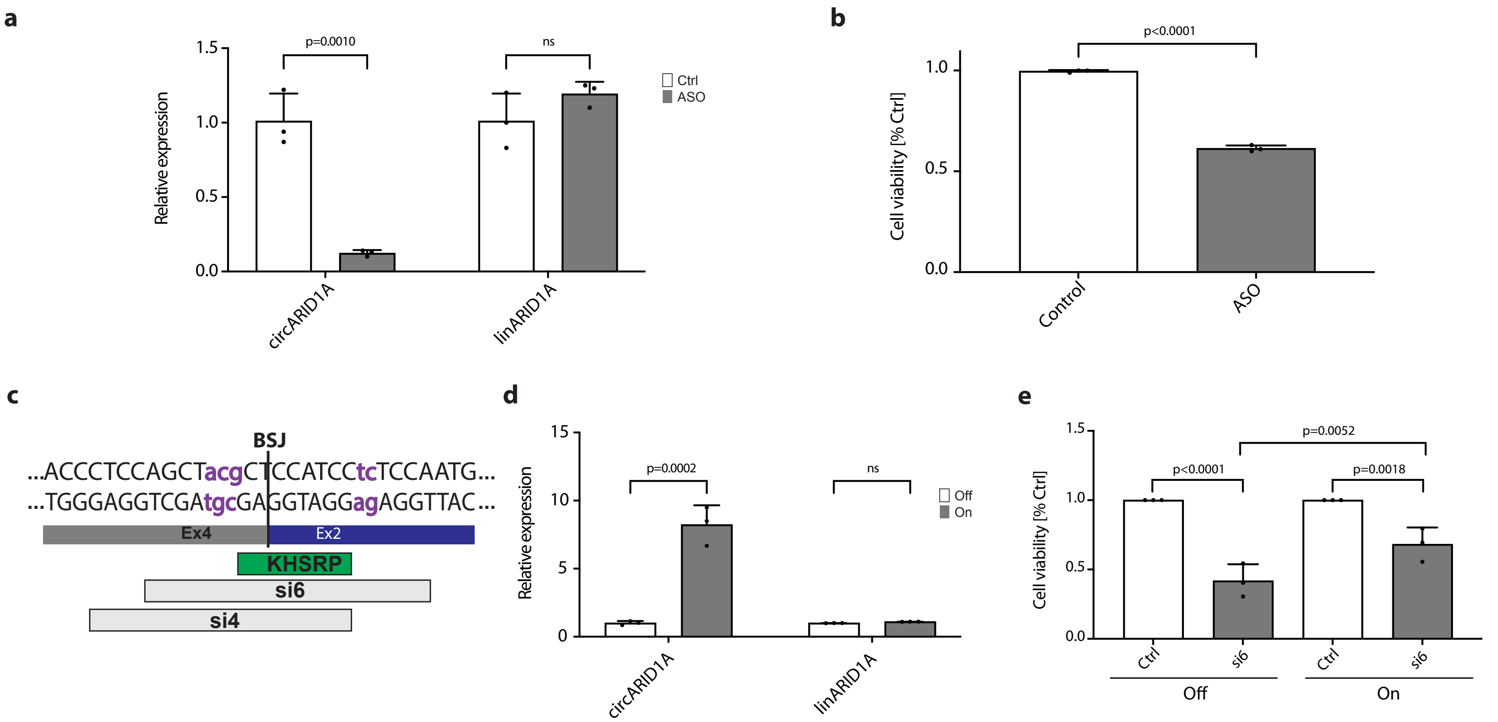
a
Relative expression
p=0.0010
ns
Ctrl
ASO
circARID1A
linARID1A
b
Cell viability [% Ctrl]
p<0.0001
Control
ASO
c
BSJ
...ACCCTCCAGCTacgCTCCATCCtcTCCAATG...
...TGGGAGGTCGAtgcGAGGTAGGagAGGTTAC...
Ex4
Ex2
KHSRP
si6
si4
d
Relative expression
p=0.0002
ns
Off
On
circARID1A
linARID1A
e
Cell viability [% Ctrl]
p=0.0052
p<0.0001
p=0.0018
Ctrl
si6
Ctrl
si6
Off
On

Figure S8| The specificity of the knockdown of circARID1A was confirmed by alternative strategies.

a, circARID1A was knocked down in IMR-5 cells by an antisense oligonucleotide (ASO) and the expression of circARID1A and *ARID1A* mRNA assessed by qRT-PCR (n=3 biologically independent experiments, Data are presented as mean ± SD, Two-way ANOVA test).

b, Cell viability was measured after ASO-mediated knockdown in IMR-5 cells (n=3 biologically independent experiments, Data are presented as mean ± SD, Two-sided unpaired Student's t-test).

c, The scheme shows the overexpression construct with the mutated back-splice junction (BSJ) of circARID1A, the binding sites of KHSRP and the two different siRNAs used for the rescue experiment with SH-EP cells. Mutated nucleotides are marked in purple.

d, The overexpression of circARID1A without (Off) or with (On) induction was validated by qRT-PCR (n=3 biologically independent experiments, Data are presented as mean ± SD, Two-way ANOVA test).

e, Cell viability after knockdown of circARID1A without (Off) of with (On) inducing the ectopic expression of the mutated circARID1A was determined (n=3 biologically independent experiments, Data are presented as mean ± SD, One-way ANOVA test). Source data are provided as a Source Data file.

Fig. S9

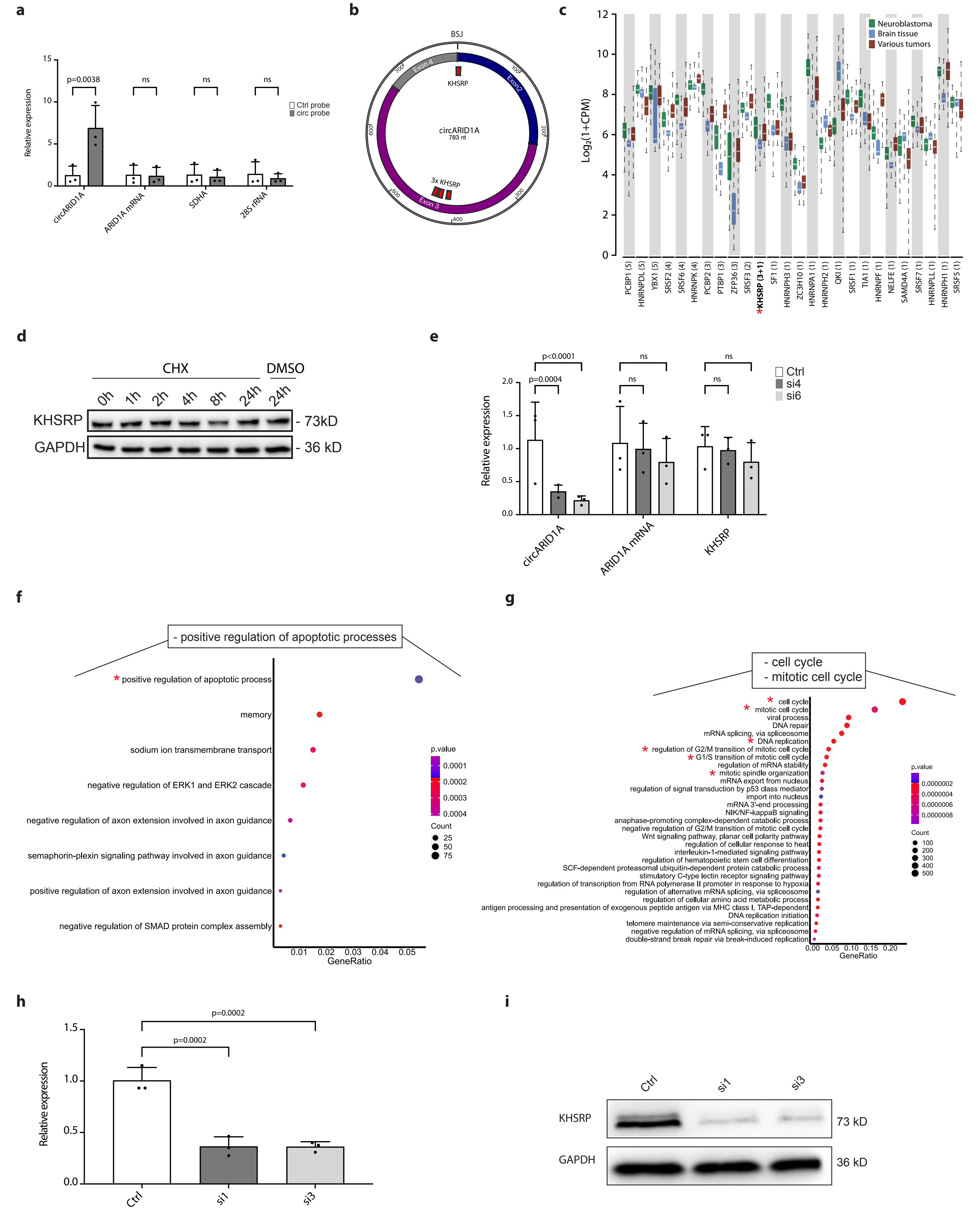

a
Relative expression
p=0.0038
ns
ns
ns
Ctrl probe
circ probe
circARID1A
ARID1A mRNA
SDHA
28S rRNA
b
BSJ
Exon 4
Exon2
KHSRP
circARID1A
783 nt
3x KHSRP
Exon 3
c
Log2(1+CPM)
Neuroblastoma
Brain tissue
Various tumors
PCBP1 (5)
HNRNPDL (5)
YBX1 (5)
SRSF2 (4)
SRSF6 (4)
HNRNPK (4)
PCBP2 (3)
PTBP1 (3)
ZFP36 (3)
SRSF3 (2)
*KHSRP (3+1)
SF1 (1)
HNRNPH3 (1)
ZC3H10 (1)
HNRNPA1 (1)
HNRNPH2 (1)
QKI (1)
SRSF1 (1)
TIA1 (1)
HNRNPF (1)
NELFE (1)
SAMD4A (1)
SRSF7 (1)
HNRNPLL (1)
HNRNPH1 (1)
SRSF5 (1)
d
CHX
DMSO
0h
1h
2h
4h
8h
24h
24h
KHSRP
GAPDH
- 73kD
- 36 kD
e
Relative expression
p<0.0001
p=0.0004
ns
ns
ns
ns
Ctrl
si4
si6
circARID1A
ARID1A mRNA
KHSRP
f
- positive regulation of apoptotic processes
* positive regulation of apoptotic process
memory
sodium ion transmembrane transport
negative regulation of ERK1 and ERK2 cascade
negative regulation of axon extension involved in axon guidance
semaphorin-plexin signaling pathway involved in axon guidance
positive regulation of axon extension involved in axon guidance
negative regulation of SMAD protein complex assembly
p.value
Count
GeneRatio
g
- cell cycle
- mitotic cell cycle
* cell cycle
* mitotic cell cycle
viral process
DNA repair
mRNA splicing, via spliceosome
* DNA replication
* regulation of G2/M transition of mitotic cell cycle
* G1/S transition of mitotic cell cycle
regulation of mRNA stability
* mitotic spindle organization
mRNA export from nucleus
regulation of signal transduction by p53 class mediator
import into nucleus
mRNA 3'-end processing
NIK/NF-kappaB signaling
anaphase-promoting complex-dependent catabolic process
negative regulation of G2/M transition of mitotic cell cycle
Wnt signaling pathway, planar cell polarity pathway
regulation of cellular response to heat
interleukin-1-mediated signaling pathway
regulation of hematopoietic stem cell differentiation
SCF-dependent proteasomal ubiquitin-dependent protein catabolic process
stimulatory C-type lectin receptor signaling pathway
regulation of transcription from RNA polymerase II promoter in response to hypoxia
regulation of alternative mRNA splicing, via spliceosome
regulation of cellular amino acid metabolic process
antigen processing and presentation of exogenous peptide antigen via MHC class I, TAP-dependent
DNA replication initiation
telomere maintenance via semi-conservative replication
negative regulation of mRNA splicing, via spliceosome
double-strand break repair via break-induced replication
p.value
Count
GeneRatio
h
Relative expression
p=0.0002
p=0.0002
Ctrl
si1
si3
i
Ctrl
si1
si3
KHSRP
GAPDH
73 kD
36 kD

Figure S9| KHSRP participates in the function of circARID1A by direct interaction.

a, Enrichment of circARID1A after pulldown in comparison to *ARID1A* mRNA and control transcripts determined by qRT-PCR (n=3 biologically independent experiments, Data are presented as mean ± SD, Two-way ANOVA test).

b, The scheme shows circARID1A and the enriched KHSRP sites determined *in silico*. The back-splice junction is marked (BSJ). The figure was created with SnapGene Viewer.

c, Expression of RNA-binding proteins (RBPs) with in silico identified motifs enriched in the circARID1A sequence (number of motifs in parenthesis) in neuroblastoma (green, n=104 biologically independent samples), brain tissue (blue, n=72 biologically independent samples) and other tumors (brown, n=86 biologically independent samples) shown as in **Figure 5a**. KHSRP (3 canonical and 1 non-canonical site) is marked in bold and by an asterisk. Data are presented as a box plot. The box plot center line, box limits and whiskers indicate the median, upper/lower quartiles and 1.5× interquartile range respectively.

d, Treatment of IMR-5 cells with cycloheximide (CHX) to block translation in comparison to a control treatment with DMSO and assessment of KHSRP and GAPDH protein levels by western blot (n=1).

e, Expression of circARID1A, *ARID1A* mRNA, and *KHSRP* after circARID1A knockdown in IMR-5 cells (n=3 biologically independent experiments, Data are presented as mean ± SD, Two-way ANOVA test).

f, The enrichment of gene ontology terms (biological process, GO terms) in upregulated genes after *KHSRP* knockdown (n=3 biologically independent experiments) was calculated. Important terms are highlighted by an asterisk.

g, Calculation of enriched GO terms in downregulated genes after *KHSRP* knockdown in IMR-5 cells (n=3 biologically independent experiments). Important terms are highlighted by an asterisk.

h, Confirmation of *KHSRP* knockdown in IMR-5 cells by qRT-PCR (n=3 biologically independent experiments, Data are presented as mean ± SD, one-way ANOVA test).

i, KHSRP abundance determined by western blot after *KHSRP* knockdown in IMR-5 cells (n=3 biologically independent experiments). Source data are provided as a Source Data file.
