# Supplementary material for: Defining the landscape of circular RNAs in neuroblastoma unveils a global suppressive function of MYCN
Source: Nat Commun. 2023 Jul 4;14:3936. doi: 10.1038/s41467-023-38747-4 (PMC10319804; doi:10.1038/s41467-023-38747-4)
Supplement: Supplementary file 2 — Description of Additional Supplementary Files [file 41467_2023_38747_MOESM2_ESM.pdf]

Description of Additional Supplementary Files

File Name: Supplementary Data 1
Description: Characteristics of patients enrolled in this study.

File Name: Supplementary Data 2
Description: circRNAs identified in neuroblastoma tumors.

File Name: Supplementary Data 3
Description: Identified circRNA hotspots.

File Name: Supplementary Data 4
Description: Genes and circRNAs differentially expressed in high-risk neuroblastomas.

File Name: Supplementary Data 5
Description: Genes and circRNAs differentially expressed after DHX9 knockdown.

File Name: Supplementary Data 6
Description: List of neuroblastoma-specific circRNAs.

File Name: Supplementary Data 7
Description: Genes differentially expressed after KHSRP knockdown.

File Name: Supplementary Data 8
Description: Genes and circRNAs differentially expressed after circARID1A knockdown.

File Name: Supplementary Data 9
Description: Oligonucleotides and antibodies used in this study.
