# Supplementary material for: Defining the landscape of circular RNAs in neuroblastoma unveils a global suppressive function of MYCN
Source: Nat Commun. 2023 Jul 4;14:3936. doi: 10.1038/s41467-023-38747-4 (PMC10319804; doi:10.1038/s41467-023-38747-4)
Supplement: Supplementary file 12 — Reporting Summary [file 41467_2023_38747_MOESM12_ESM.pdf]

nature portfolio

Corresponding author(s): Johannes H. Schulte, Steffen Fuchs

Last updated by author(s): May 10, 2023

Reporting Summary

Nature Portfolio wishes to improve the reproducibility of the work that we publish. This form provides structure for consistency and transparency in reporting. For further information on Nature Portfolio policies, see our Editorial Policies and the Editorial Policy Checklist.

Statistics

For all statistical analyses, confirm that the following items are present in the figure legend, table legend, main text, or Methods section.

n/a	Confirmed	
☐	☒	The exact sample size (*n*) for each experimental group/condition, given as a discrete number and unit of measurement
☐	☒	A statement on whether measurements were taken from distinct samples or whether the same sample was measured repeatedly
☐	☒	The statistical test(s) used AND whether they are one- or two-sided<br>*Only common tests should be described solely by name; describe more complex techniques in the Methods section.*
☐	☒	A description of all covariates tested
☐	☒	A description of any assumptions or corrections, such as tests of normality and adjustment for multiple comparisons
☐	☒	A full description of the statistical parameters including central tendency (e.g. means) or other basic estimates (e.g. regression coefficient) AND variation (e.g. standard deviation) or associated estimates of uncertainty (e.g. confidence intervals)
☐	☒	For null hypothesis testing, the test statistic (e.g. *F*, *t*, *r*) with confidence intervals, effect sizes, degrees of freedom and *P* value noted<br>*Give P values as exact values whenever suitable.*
☒	☐	For Bayesian analysis, information on the choice of priors and Markov chain Monte Carlo settings
☒	☐	For hierarchical and complex designs, identification of the appropriate level for tests and full reporting of outcomes
☐	☒	Estimates of effect sizes (e.g. Cohen's *d*, Pearson's *r*), indicating how they were calculated

Our web collection on statistics for biologists contains articles on many of the points above.

Software and code

Policy information about availability of computer code

Data collection

- qPCR: StepOne Plus Software (v2.3, Applied Biosystems)
- Real-Time proliferaton measurement with the IncuCyte® S3 Live Cell Systems (software v2019b Rev2, Essen Biosciences)
- Cell viability measurement with Glomax Multi + Detection Systems (software v3.1.3, Promega)
- Fluorescence microscopy aquisition with NIS-Elements Imaging Software (v5.02.00, Nikon, Minato, Japan)
- Flow cytometry: BD LSR Fortessa X-20 flow cytometer (software BD FACS Diva software v8.0.1, BD Biosciences, Franklin Lakes, NJ, USA)
- Nanopore sequencing: MinION MK1C sequencer (Oxford Nanopore; operating software for data aquisition MinKNOW v22.058, further information can be found in our detailed published protocol https://dx.doi.org/10.17504/protocols.io.rm7vzy8r4lx1/v2

Data analysis

Scripts and code used for sequencing data analysis in this study are well established and fully referenced in the methods section. We further deposited codes and scripts to reproduce our analysis on Github under the following link: http://github.com/josc3006/circRNA_NB.

bcl2fastq (v2.20)
FastQC (v0.11.7)
BWA-MEM (v0.7.10, v0.7.15 and v0.7.17-r1188)
samblaster 53 (v0.1.24).
Control-FREEC 54, (v11.0)
STAR (v2.7.1a)
featureCounts (v1.5.1)
kallisto (v0.44.0)
CIRI2 (v2.0.6)
DESeq2 (v.1.32.0)

edgeR (v.3.34.1)
SUPPA2 (v.2.3)
ProliferativeIndex CRAN package (v1.0.1)
circlize (v0.4.5)
ComplexHeatmap (v1.10.2 and v2.6.2)
VennDiagram (v1.6.20).
ggplot2 (v3.0.0)
PANTHER (v14)
BBMap (v38.58)
Picard (v2.20.4)
deepTools (v3.3.0)
IGV (v2.3.93)
MACS2 (v2.1.2)
Phantompeakqualtools (v1.2.1)
growthrates' (https://CRAN.R-project.org/package=growthrates) (v0.8.2)
DescTools' (v0.99.40)
MaxQuant software (v1.6.3.4)
Perseus software package (v. 1.6.2.1)
MONSDA (v1.0.0, doi:10.31219/osf.io/jeqgr)
RepeatMasker (v4.1.4)
bedtools (v2.30.0)
FlowJo (v10.8)
cutadapt (v3.4)
CIRI-Long (v1.0.3)
minimap2 (v2.19)
GraphPad Prism 9
Microsoft Excel 2016
ImageJ (v1.46m)
Adobe Lightroom (V.10.0)
SnapGene Viewer (v6.0.7)

For manuscripts utilizing custom algorithms or software that are central to the research but not yet described in published literature, software must be made available to editors and reviewers. We strongly encourage code deposition in a community repository (e.g. GitHub). See the Nature Portfolio guidelines for submitting code & software for further information.

Data

Policy information about availability of data

All manuscripts must include a data availability statement. This statement should provide the following information, where applicable:

- Accession codes, unique identifiers, or web links for publicly available datasets
- A description of any restrictions on data availability
- For clinical datasets or third party data, please ensure that the statement adheres to our policy

The RNA sequencing and ChIP sequencing data used in this study are publicly available in the NCBI Gene Expression Omnibus under accession codes GSE102741 (https://www.ncbi.nlm.nih.gov/geo/query/acc.cgi?acc=GSE102741)95, GSE71315 (https://www.ncbi.nlm.nih.gov/geo/query/acc.cgi?acc=GSE71315)97, GSE62563 (https://www.ncbi.nlm.nih.gov/geo/query/acc.cgi?acc=GSE62563)98, GSE95277 (https://www.ncbi.nlm.nih.gov/geo/query/acc.cgi?acc=GSE95277)99, GSE78785 (https://www.ncbi.nlm.nih.gov/geo/query/acc.cgi?acc=GSE78785)100, GSE77509 (https://www.ncbi.nlm.nih.gov/geo/query/acc.cgi?acc=GSE77509)101, GSE77661 (https://www.ncbi.nlm.nih.gov/geo/query/acc.cgi?acc=GSE77661)102, GSE80151 (https://www.ncbi.nlm.nih.gov/geo/query/acc.cgi?acc=GSE80151)56, GSE94782 (https://www.ncbi.nlm.nih.gov/geo/query/acc.cgi?acc=GSE94782)82, GSE138295 (https://www.ncbi.nlm.nih.gov/geo/query/acc.cgi?acc=GSE138295)82, GSE64425 (https://www.ncbi.nlm.nih.gov/geo/query/acc.cgi?acc=GSE64425)38, from the ENCODE consortium under accession numbers ENCSR000AEW (https://www.encodeproject.org/experiments/ENCSR000AEW/), ENCSR000AFD (https://www.encodeproject.org/experiments/ENCSR000AFD), ENCSR000AFE (https://www.encodeproject.org/experiments/ENCSR000AFE), ENCSR000AEX (https://www.encodeproject.org/experiments/ENCSR000AEX), ENCSR000AEY (https://www.encodeproject.org/experiments/ENCSR000AEY), ENCSR000AFJ (https://www.encodeproject.org/experiments/ENCSR000AFJ)96 and from the European Genome-phenome Archive under accession number EGAD00001003279 (https://ega-archive.org/datasets/EGAD00001003279)39 after approval by the data access committee.
The genome references hg38 (https://ftp.ensembl.org/pub/release-109/fasta/homo_sapiens/dna/Homo_sapiens.GRCh38.dna.alt.fa.gz), hg19 (https://ftp.ensembl.org/pub/grch37/current/fasta/homo_sapiens/dna/Homo_sapiens.GRCh37.dna.alt.fa.gz) and mm10 (ftp://ftp.ensembl.org/pub/release-102/fasta/mus_musculus/dna/) were downloaded from the ensemble website (https://ensemblgenomes.org) and the transcriptome annotation Gencode v30 from the gencode website (https://www.gencodegenes.org/human/release_30.html).
The mass spectrometry data generated in this study have been publicly deposited in the PRIDE repository under accession code PXD026053 (https://proteomecentral.proteomexchange.org/cgi/GetDataset?ID=PXD026053). The RNA sequencing and Whole Genome Sequencing data of neuroblastoma patients are deposited in the European Genome-phenome Archive under accession codes EGAS00001004022 (https://ega-archive.org/studies/EGAS00001004022), EGAS00001005604 (https://ega-archive.org/studies/EGAS00001005604). This dataset from neuroblastoma patients is available under restricted access due to data privacy laws. Access to the EGA archive dataset is obtained by formal application to the Data Access Committee (DAC, https://ega-archive.org/dacs/EGAC00001002310) at johannes.schulte@charite.de. The DAC will honor legitimate requests for sequencing data from researchers as necessary for conducting methodologically sound research for precise projects. The DAC requires users/applicants to sign a Data Access Agreement (DAA), which details the terms and conditions of use for each dataset, for example to use the data only for the project that was applied for, and to not share the data with other parties. The DAC will respond to requests within 2 weeks and provide access to the data within 4 weeks. The data will be made available for 12 months, once the DAA is signed by both sides. Further RNA sequencing and Nanopore data have been publicly deposited at the NCBI Gene Expression Omnibus under accession numbers GSE174571 (https://www.ncbi.nlm.nih.gov/geo/query/acc.cgi?acc=GSE174571), GSE174572 (https://www.ncbi.nlm.nih.gov/geo/query/acc.cgi?acc=GSE174572), GSE174708 (https://www.ncbi.nlm.nih.gov/geo/query/acc.cgi?acc=GSE174708), GSE181561 (https://www.ncbi.nlm.nih.gov/geo/query/acc.cgi?acc=GSE181561), GSE223105 (https://www.ncbi.nlm.nih.gov/geo/query/acc.cgi?acc=GSE223105).
The remaining data are available within the Article, Supplementary Information or Source Data file. Further information and requests for resources and reagents should be directed to and will be fulfilled by the Lead Contact, Johannes H. Schulte, johannes.schulte@charite.de.

Human research participants

Policy information about studies involving human research participants and Sex and Gender in Research.

Reporting on sex and gender	Neuroblastoma is more common in boys than in girls, with a ratio of 1.4:1, we tried to take this into account when planning this study (ref. to: Kaatsch, et al.: German Childhood Cancer Registry – Annual Report 2017). However, due to limited sample availability of this rare disease (incidence 10.5 per 1 million children, Park et al., 2010) the actual male:female ratio in our cohort is 1.86:1, which brings a light bias. Sex was assigned. We do not have information on gender. Patient characteristics are reported in supplementary file 1.<br>Concerning the cell lines we used, we achieved an almost equal distribution: 5 cell lines were male, and 7 are female. Of note, 3 female cell lines (SH-SY5Y and SH-EP are clones from SK-N-SH) and 2 male cell lines are clones (IMR-5 and IMR-5/75 are clones from IMR-32) derived from another cell line.<br>Our research findings are not limited to only one sex or gender.
Population characteristics	see methods section:<br>Patients were enrolled in the German neuroblastoma clinical trials (NB90, NB97, NB2004) or the German Neuroblastoma Registry (NB Registry 2016). Patients were registered and treated according to trial protocols from the German Society of Pediatric Oncology and Hematology (GPOH). Primary tumor samples from initial biopsies were collected by the German Neuroblastoma Biobank (Cologne) from clinical trial sites. The German Neuroblastoma Biobank provided total RNA isolated from primary tumor samples collected from 104 patients diagnosed with neuroblastoma, of which 36 were female, 67 male and 1 patient for that sex information was not available. Gender information were not available for the patients. The average age of patients was 2.4 years.
Recruitment	Selection was based on tumor sample availability. The cohort consists of all neuroblastoma risk-groups.
Ethics oversight	This study was conducted in accordance with the Declaration of Helsinki and Good Clinical Practice, and informed consent was obtained from all patients and/or their guardians. Collection and use of patient samples was approved by the institutional review boards of Charité Universitätsmedizin Berlin and the University of Cologne Medical Faculty within the trial and registry, respectively.

Note that full information on the approval of the study protocol must also be provided in the manuscript.

Field-specific reporting

Please select the one below that is the best fit for your research. If you are not sure, read the appropriate sections before making your selection.

☒ Life sciences ☐ Behavioural & social sciences ☐ Ecological, evolutionary & environmental sciences

For a reference copy of the document with all sections, see nature.com/documents/nr-reporting-summary-flat.pdf

Life sciences study design

All studies must disclose on these points even when the disclosure is negative.

Sample size	Due to the exploratory study design, no sample size calculation could be made prior to acquiring neuroblastoma WGS/RNA-seq data. Sample size was therefore determined by availability of patient samples. Matched WGS was only available of 64 of 104 of our neuroblastoma patients. Further sample size was given by the sample size of the public data analyzed. For ChIP-seq we analyzed 3 different MYCN-amplified and 1 non-amplified neuroblastoma cell line. For further characterization of circARID1A we chose 2 MYCN-amplified and 2 non MYCN-amplified neuroblastoma cell line.
Data exclusions	For patients CB2009, CB3024 and CB3042 the total RNA-seq data had to be excluded due to low library quality.<br>For patients CB3011 and 3042 not enough RNA of sufficient quality was received from the biobank for RNA-seq library construction.<br>Patient CB3036 was excluded due to heterogenous MYCN amplification (most cells without MYCN amplification as determined by DNA-FISH by the Neuroblastoma Biobank Cologne).
Replication	The RNA-seq and WGS of our neuroblastoma cohort was not acquired in replicates, but the cohort itself is relatively large taken into account that the tumor entitiy neuroblastoma is rare. We further used published sequencing data that had not been acquired in replicates.<br>We validated our findings obtained in our neuroblastoma cohort by re-analyzing published data from 2 independent neuroblastoma cohorts (mass spectrometry and RNA-seq). Further, we analyzed data of a different tumor entity with MYCN aberrations, medulloblastoma, to increase robustness of our study (RNA-seq and ChIP-seq).<br>Cell models with modifiable MYCN expression were used to reproduce the findings obtained in neuroblastoma tumors.<br>Nanopore long-read sequencing was performed in 6 different neuroblastoma cell lines (MYCN amplified and not). All replicates were succesful in reproducing the findings.<br><br>To assess effects of the circARID1A knock-down, four independent cell lines (2 with, 2 without MYCN amplification) as well as two independent siRNAs were used. The knockdown was further verified by an independent treatment with antisense oligonucleotides. The specificity of the knockdown was validated by a rescue experiment.

Interaction of circARID1A and KHSRP was detected by pulldown and validated by an orthogonal strategy (RNA-IP) to increase robustness.

All measures taken to increase reproducibility were described in detail in our methods section.

Randomization	Randomization was not applicable as patients were not allocated into experimental groups.<br>For cell culture-based experiments, cells were split from the same batch of cells and randomly divided for each treatment in each biological replicate.
Blinding	Blinding was not applicable as patients were not allocated into experimental groups.<br>For cell culture-based experiments, the investigators were not blinded to allocation during experiments and outcome assessment, as proper controls were already included during the experimental design.

Reporting for specific materials, systems and methods

We require information from authors about some types of materials, experimental systems and methods used in many studies. Here, indicate whether each material, system or method listed is relevant to your study. If you are not sure if a list item applies to your research, read the appropriate section before selecting a response.

Materials & experimental systems

n/a	Involved in the study	
☐	☒	Antibodies
☐	☒	Eukaryotic cell lines
☒	☐	Palaeontology and archaeology
☒	☐	Animals and other organisms
☐	☒	Clinical data
☒	☐	Dual use research of concern

Methods

n/a	Involved in the study	
☒	☐	ChIP-seq
☐	☒	Flow cytometry
☒	☐	MRI-based neuroimaging

Antibodies

Antibodies used

All antibodies also are listed in supplementary file 9.

MYCN (Santa Cruz, sc-53993, lot C1315, WB, dilution 1:200)
GAPDH (FL-335) (Santa Cruz, sc-25778, lot A0515, WB, dilution 1:1000)
RNA HelicaseA (DHX9, EPR13521) (Abcam, ab183731, lot 9I10I20, WB, dilution 1:1000)
RNA HelicaseA (DHX9) (Novus Biologicals, NB110-40579, IP, used amount for IP: 5µg)
KHSRP (Bethyl Lab, A302-022A, lot 1, IP, used amount for IP:5µg)
KHSRP (EPR9865) (Abcam, ab140648, lot GR103988-8, WB, dilution 1:1000)
HuR (3A2, ELAVL1, pan-ELAVL) (Santa Cruz, sc-5261, lot A0620, WB, dilution 1:1000, used amount for IP: 5µg)
Non-specific IgG control (Santa Cruz, sc-2025, lot J2015, used amount for IP: 5µg)
Anti-mouse (Dianova, 115-035-146 , lot 136993, WB, dilution 1:5000)
Anti-rabbit (Dianova, 111-005-003, lot 137093, WB, dilution 1:5000)
Veriblot for IP Detection reagent (used as secondary antibody for WB after IP) (Abcam, ab131366, lot GR3364349-1, WB, dilution 1:1000)

Validation

All antibodies were validated by the manufacturer for the respective use:

MYCN: WB (https://www.scbt.com/p/n-myc-antibody-b8-4-b)
GAPDH (FL-335): WB (https://www.scbt.com/p/gapdh-antibody-fl-335?requestFrom=search)
RNA HelicaseA (DHX9, EPR13521): WB (https://www.abcam.com/rna-helicase-a-antibody-epr13521-ab183731.html)
RNA HelicaseA (DHX9): IP (https://www.novusbio.com/products/rna-helicase-a-antibody_nb110-40579)
KHSRP: IP (https://www.bethyl.com/product/A302-022A/KSRP+Antibody)
KHSRP (EPR9865): WB (https://www.abcam.com/KHSRP-antibody-EPR9865-ab140648.html)
HuR (3A2, ELAVL1, pan-ELAVL): WB, IP (https://www.scbt.com/p/hur-antibody-3a2)
Non-specific IgG control: IP (https://www.scbt.com/p/normal-mouse-igg?requestFrom=search)
Anti-mouse: WB (https://www.dianova.com/shop/115-035-146-ziege-igg-anti-maus-igg-hl-hrpo-minx-hubohorbsw/)
Anti-rabbit: WB (https://www.dianova.com/shop/111-005-003-ziege-igg-anti-kaninchen-igg-hl-unkonj-minx-keine/)
Veriblot for IP Detection reagent (used as secondary antibody for WB after IP): WB (https://www.abcam.com/veriblot-for-ip-detection-reagent-hrp-ab131366.html)

Eukaryotic cell lines

Policy information about cell lines and Sex and Gender in Research

Cell line source(s)

see methods:
The human neuroblastoma cell lines SK-N-BE(2)C (CRL-2268) and SK-N-FI (CRL-2142) was obtained from the American Type Culture Collection (ATCC, Manassas, VA, USA) and the cell lines Kelly (ACC-355), SH-SY5Y (ACC-209), LAN-5 (ACC-673) were obtained from the German Collection of Microorganisms and Cell Cultures GmbH (DSMZ, Braunschweig, Germany). IMR5/75

was kindly provided by F. Westermann (German Cancer Research Center, Heidelberg, Germany). IMR-5, SH-EP, SK-N-AS, SK-N-SH and GI-ME-N were kindly provided by A. Schramm (Medizinische Fakultät, Universitätsklinikum Essen, Essen, Germany). LS cells were kindly provided by M. Fischer (University of Cologne Medical Faculty). The MYCN-inducible cell model was previously generated from the SK-N-AS neuroblastoma cell line (kindly provided by A. Schramm, Medizinische Fakultät, Universitätsklinikum Essen, Essen, Germany; Tjaden, 2020). IMR-5/75 cells harboring an inducible expression system for an shRNA targeting MYCN were previously generated (Muth, 2010; kindly provided by F. Westermann, DKFZ, Heidelberg, Germany).

Authentication

Cell lines were authenticated by short tandem repeat DNA typing (Idexx Bioresearch, or Multiplexion)

Mycoplasma contamination

All cells tested negative for mycoplasma using the MycoAlert Mycoplasma Detection Kit (Lonza).

Commonly misidentified lines
(See ICLAC register)

None of the used cell lines (SK-N-SH, SH-SY5Y, GI-ME-N, SH-EP, IMR-5, IMR-5/75, KELLY, LAN-5, SK-N-BE(2)C, SK-N-FI, SK-N-AS and LS) is a commonly misidentified line.

Clinical data

Policy information about clinical studies
All manuscripts should comply with the ICMJE guidelines for publication of clinical research and a completed CONSORT checklist must be included with all submissions.

Clinical trial registration

*** This was not a clinical trial. ***

Study protocol

We analyzed tissue and blood samples from patients who participated in the clinical trials NB90, NB97, NB2004 and German neuroblastoma registry (NB Registry 2016), studies conducted by the German Society of Pediatric Oncology and Hematology (GPOH).

NB90: https://clinicaltrials.gov/ct2/show/NCT00002802
NB97: https://clinicaltrials.gov/ct2/show/NCT00017225?term=NB97&cntry=DE&draw=2&rank=1
NB2004: https://www.kinderkrebsinfo.de/e1676/e9032/e68518/e206421/download7673/NB_2004_1.00_komprimiert__ger.pdf
NB2016 registry: https://www.kinderkrebsinfo.de/health_professionals/clinical_trials/pohkinderkrebsinfotherapiestudien/nb_registry_2016/index_eng.html

Data collection

Specimens and clinical data were archived and made available by Charité-Universitätsmedizin Berlin or the German Neuroblastoma Biobank (University of Cologne Medical Faculty) of the GPOH.

Outcomes

The only reported outcome for the patients were overall survival events (ocurrence of death) and event-free survival events (occurrence of relapse).

Flow Cytometry

Plots

Confirm that:

☒ The axis labels state the marker and fluorochrome used (e.g. CD4-FITC).

☒ The axis scales are clearly visible. Include numbers along axes only for bottom left plot of group (a 'group' is an analysis of identical markers).

☒ All plots are contour plots with outliers or pseudocolor plots.

☒ A numerical value for number of cells or percentage (with statistics) is provided.

Methodology

Sample preparation

To study apoptosis induction by cARID1A knockdown in different neuroblastoma cell lines, APC-AnnexinV (Biolegend, San Diego, CA, USA) and 7-AAD viability solution (Thermo Fisher) were used according to the manufacturer. Cells were harvested using Accutase (Sigma-Aldrich) and counted to determine the cell number and viability.
To study GFP positive cells in the DHX9 reporter assay, cells were harvested similarily using Accutase (Sigma-Aldrich) and counted to determine the cell number and viability.

Instrument

BD LSR Fortessa X-20 flow cytometer (BD Biosciences, Franklin Lakes, NJ, USA)

Software

Data aquisition: BD FACS Diva software (v8.0.1). Data analysis: FlowJo (v10.8)

Cell population abundance

After eliminating debris and doublets all cells were considered for the analysis , which were usually more than 80% of cells.

Gating strategy

AnnexinV assay:
From FSC-A/SSC-A, cell debris was eliminated. From SSC-H/SSC-A single cells were selected. Apoptotic cells were identified by Annexin V-APC-A positive staining.
DHX9 reporter assay:
From FSC-A/SSC-A, cell debris was eliminated. From SSC-H/SSC-A single cells were selected. In wild type cells, from FSC-A and FITC-A a gate was selected so that almost no positive events were detected. The same gate was used for the cells harboring the expression plasmid to detect GFP positive cells.

☒ Tick this box to confirm that a figure exemplifying the gating strategy is provided in the Supplementary Information.
